# To Be or Not To Be Polar: The Ferroelectric and Antiferroelectric Nematic Phases

**DOI:** 10.1021/acsomega.3c05884

**Published:** 2023-09-18

**Authors:** Ewan Cruickshank, Paulina Rybak, Magdalena M. Majewska, Shona Ramsay, Cheng Wang, Chenhui Zhu, Rebecca Walker, John M. D. Storey, Corrie T. Imrie, Ewa Gorecka, Damian Pociecha

**Affiliations:** † Department of Chemistry, School of Natural and Computing Sciences, University of Aberdeen, Aberdeen AB24 3UE, U.K.; ‡ Faculty of Chemistry, University of Warsaw, ul. Zwirki i Wigury 101, 02-089 Warsaw, Poland; § Advanced Light Source, Lawrence Berkeley National Laboratory, 1 Cyclotron Road, Berkeley, California 94720, United States

## Abstract

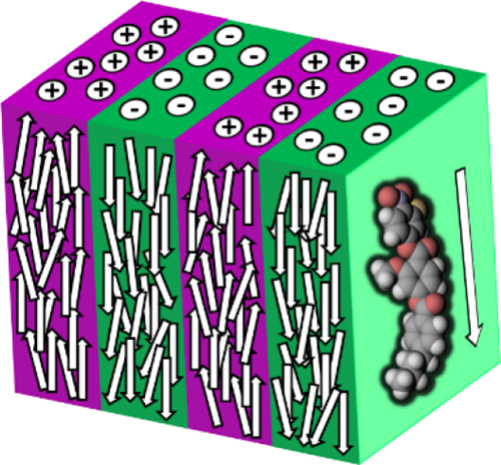

We report two new series of compounds that show the ferroelectric nematic, N_F_, phase in which the terminal chain length is varied. The longer the terminal chain, the weaker the dipole–dipole interactions of the molecules are along the director and thus the lower the temperature at which the axially polar N_F_ phase is formed. For homologues of intermediate chain lengths, between the non-polar and ferroelectric nematic phases, a wide temperature range nematic phase emerges with antiferroelectric character. The size of the antiparallel ferroelectric domains critically increases upon transition to the N_F_ phase. In dielectric studies, both collective (“ferroelectric”) and non-collective fluctuations are present, and the “ferroelectric” mode softens weakly at the N–N_X_ phase transition because the polar order in this phase is weak. The transition to the N_F_ phase is characterized by a much stronger lowering of the mode relaxation frequency and an increase in its strength, and a typical critical behavior is observed.

## Introduction

Ferroelectric materials have a spontaneous reversible electric polarization and show piezoelectric and pyroelectric properties ensuring their widespread use in leading-edge electronics such as actuators, sensors, and memory elements.^1,2^ In a liquid crystal, the switching of the electric polarization is coupled with the elastic or optical properties of the material, and this is highly desirable for applications in soft optoelectronic devices.^3^ Liquid crystalline improper ferroelectric phases have been known for decades; ferroelectric smectic phases have been studied since the 1970s^4^ and ferroelectric columnar phases since the 1990s.^5^ It has been shown that due to the competing interactions within these phases, their structures are often complex, and only a relatively small number of ferroelectric, antiferroelectric, and ferrielectric phases have been found.^6^ Furthermore, these phases have found only very limited commercialization in, for example, liquid crystal on silicon (LCoS) displays. Their wider application potential has failed to be realized mainly due to the challenge of producing defect-free large-area samples.

Recently, a polar nematic phase was discovered^7,8^ and later assigned as the ferroelectric nematic phase, N_F_.^9^ This is the least ordered polar liquid crystalline phase. In the conventional nematic phase, N, the rod-like molecules more or less align in a common direction known as the director described by a unit vector, **n**, whereas their centers of mass are distributed randomly such that the phase has a fluid character. The director possesses inversion symmetry, i.e., **n = −n**, and so, the phase is non-polar. In the N_F_ phase, however, the inversion symmetry is broken, i.e., **n** ≠ **–n**, and the phase becomes polar. It appears that this polar N_F_ phase, in contrast to the previously studied smectic and columnar phases, is a proper ferroelectric phase, in which the polar order is induced due to dipole–dipole interactions and the polarization is found along the director.^9^ The high fluidity of the N_F_ phase combined with its polar properties immediately caught the attention of scientists around the world due to not only its huge application potential but also its fundamental significance as a spontaneously ferroelectric fluid. The N_F_ phase became one of the hottest topics in liquid crystal research.^10−34^ Owing to the fluid nature of the N_F_ phase, a uniform polarization direction can be obtained in large areas—a key to realizing its application potential.^35^ However, the question arises whether the competitive interactions that drive the formation of the N_F_ phase can also lead to other complex structures as is the case with the improper ferroelectric liquid crystal phases.

**Figure 1:**
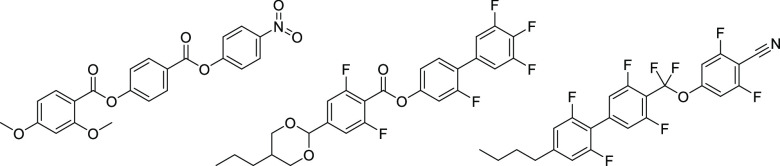
Molecular structures of the archetypal ferroelectric nematogens: (left) RM734, (middle) DIO, and (right) UUQU-4-N.

To date, there have been some 200 mesogens reported, which exhibit the N_F_ phase, but in general, these materials are designed using three archetypal architectures which appear to have somewhat similar properties despite having differing chemical structures (Figure 1).^8,11,36^ These materials all have a large longitudinal dipole moment giving strong dipole–dipole interactions and also possess some degree of lateral bulk, which is thought to inhibit the anti-parallel correlations between molecules.^37,38^

**Figure 2:**
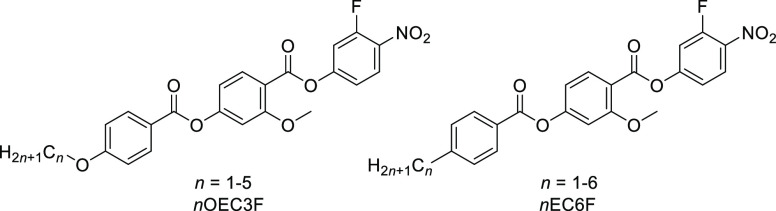
Molecular structures of (left) the *n*OEC3F and (right) the *n*EC6F series, where *n* refers to the number of carbon atoms in the terminal chain.

We report here two homologous series based on RM734^36^ (Figure 2), which both have strong longitudinal dipole moments (∼11–12 D), but in which the lateral methoxy group has been moved from the terminal to the central phenyl ring and a hydrogen *ortho* to the terminal nitro group is replaced by a fluorine atom. The series differ in the nature of the terminal chain; the *n*OEC3F series contains an alkyloxy chain and the *n*EC6F series an alkyl chain. For both series, we report the change in behavior on extending the length of the terminal chain. A detailed description of the preparation of both these series, including the structural characterization data for all intermediates and final products, is provided in the Supporting Information.

## Experimental Section

### Birefringence

The optical retardation was measured with a setup consisting of a photoelastic modulator (PEM-90, Hinds), a halogen lamp (Hamamatsu LC8) equipped with a set of narrow bandpass filters as a light source, and a photodiode (FLC Electronics PIN-20). The measured intensity of the transmitted light was de-convoluted with a lock-in amplifier (EG&G 7265) into 1f and 2f components to yield a retardation induced by the sample. Glass cells with a thickness of 1.6 μm and surfactant assuring planar anchoring condition were used.

### Dielectric Measurements

The complex dielectric permittivity was measured in the 1 Hz–10 MHz frequency (f) range using a Solartron 1260 impedance analyzer. The material was placed in glass cells with ITO or Au electrodes (and no polymer alignment layer to avoid the influence of the high capacitance of a thin polymer layer) and thickness ranging from 5 to 10 microns. The relaxation frequency, *f*_r_, and dielectric strength of the mode, Δε, were evaluated by fitting the complex dielectric permittivity to the Cole–Cole formula.

### X-ray Diffraction (XRD) Studies

XRD studies were performed at the Advanced Light Source, Lawrence Berkeley National Laboratory. Diffraction at a small angle range was carried out on the SAXS beam line (7.3.3) at the energy of incident beam 10 keV. Samples were prepared in thin-walled glass capillaries or placed on a heating plate as droplets. The scattering intensity was recorded using the Pilatus 2 M detector, placed at the distance 2575 mm from the sample. The resonant X-ray scattering was performed on the soft X-ray beam line (11.0.1.2). The energy of the incident beam was tuned to the K-edge of carbon absorption (283 eV). Samples with a thickness lower than 1 μm were placed on a transmission electron microscopy grid. The scattering intensity was recorded using the Princeton PI-MTE CCD detector.

### Molecular Modeling

The geometric parameters of the *n*OEC3F and *n*EC6F series were calculated with quantum mechanical density functional theory (DFT) calculations using Gaussian09 software.^39^ Optimization of the molecular structures was carried out at the B3LYP/6-31G(d) level of theory. A frequency check was used to confirm that the minimum energy conformation found was an energetic minimum. Visualizations of electronic surfaces and ball-and-stick models were generated from the optimized geometries using the GaussView 5 software, specifically the electronic surfaces were calculated using the cubegen utility in GaussView. Visualizations of the space-filling models were produced post-optimization using the QuteMol package.^40^

### Polarized Light Optical Microscopy

Optical studies were performed by using a Zeiss Axio Imager A2m polarizing light microscope, equipped with a Linkam heating stage or using an Olympus BH2 polarizing light microscope equipped with a Linkham TMS 92 hot stage. Samples were prepared in commercial cells (AWAT) of various thicknesses (1.5–20 μm) with ITO electrodes and planar alignment or in commercial cells purchased from INSTEC with a cell thickness of 2.9–3.5 μm and also planar alignment. The optical microscopic image was analyzed (director field and birefringence) with the ABRIO system.

### Differential Scanning Calorimetry

The phase behavior of the materials was studied by differential scanning calorimetry (DSC) performed using Mettler Toledo DSC1 or DSC3 differential scanning calorimeters equipped with TSO 801RO sample robots and calibrated using indium and zinc standards. Heating and cooling rates were 10 °C min^–1^, with a 3 min isotherm between either heating or cooling, and all samples were measured under a nitrogen atmosphere. The enantiotropic transition temperatures and associated enthalpy changes were extracted from the heating traces, whereas the monotropic transition temperatures and associated enthalpy changes were extracted from the cooling traces.

## Results and Discussion

**Figure 3:**
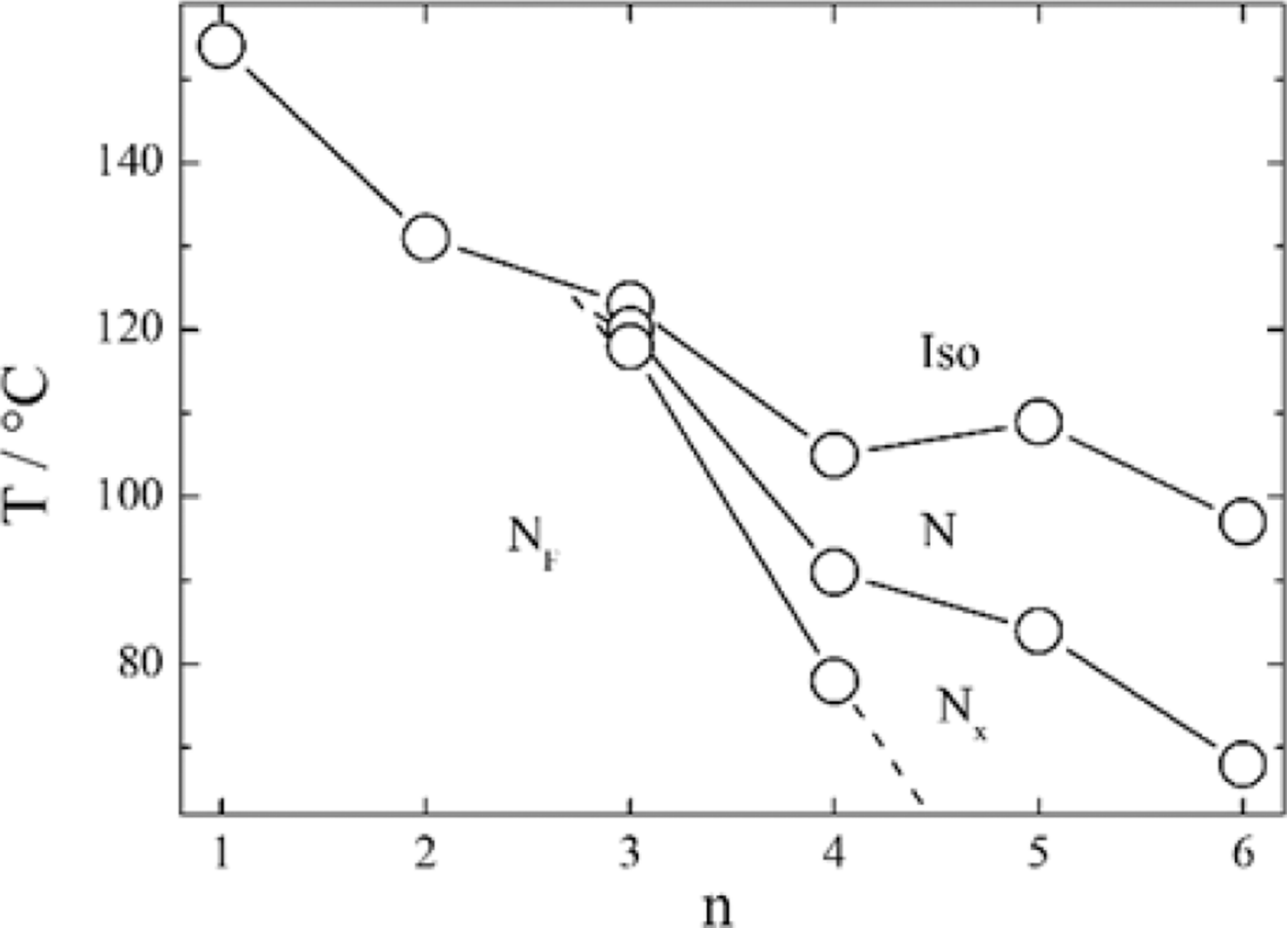
Phase diagram for the *n*EC6F series of ferroelectric nematogens with a molecular structure shown in Figure 2. The conventional non-polar nematic phase is represented by N, the antiferroelectric nematic phase by N_X_, and the polar ferroelectric nematic phase by N_F_.

We have shown previously that increasing the length of a lateral alkyloxy chain for RM734-type materials destabilizes the ferroelectric properties.^21,30,31,33^ However, the N–N_F_ phase transition temperature decreases less than the Iso-N transition temperature, such that for most homologues in which there is a fluorine atom *ortho* to the terminal nitro group, a direct Iso-ferroelectric nematic phase transition is observed. In contrast, here we observed that increasing the length of the terminal alkyl chain only weakly affects the clearing temperatures, whereas the stability of the ferroelectric N_F_ phase strongly decreases in favor of the N and intermediate N_X_ phases (Figure 3). For the *n*EC6F series, when *n* = 1 and 2, a direct Iso-N_F_ transition is seen, for *n* = 3 and 4, the sequence Iso-N-N_X_-N_F_ is observed, and for homologues *n* = 5 and 6, an Iso-N-N_X_ phase sequence is found. The longest homologues crystallized close to room temperature but without first entering the N_F_ phase (Figure 3). Such phase behavior is expected given that increasing the length of the terminal alkyl chain decreases the dipole–dipole interactions along the director, and thus, the tendency to form an axially ferroelectric arrangement of dipole moments diminishes. The stabilization of the N_X_ phase over a broad temperature range for the *n* = 5 and 6 homologues offers the possibility for a detailed characterization of this phase. This is particularly important since the structure of the N_X_ phase is still under debate. Chen et al. suggested that the phase has only short-range order regarding molecular positions but shows a regular array of antiferroelectric domains along the direction perpendicular to the director.^41^ To date, the structure of the N_X_ phase (referred to by Chen et al. as SmZ_A_) was confirmed by XRD studies in only a single compound, namely, DIO (Figure 1).

**Figure 4:**
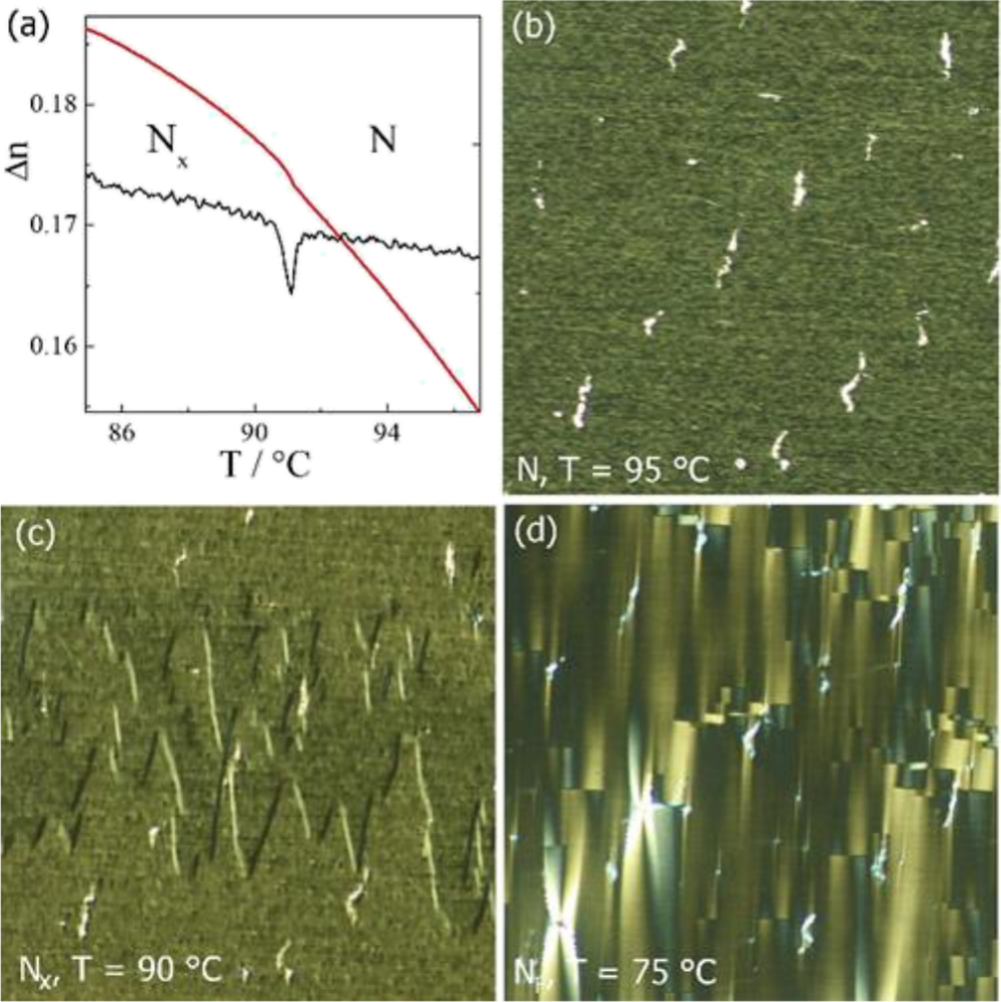
(a) Optical birefringence (red line) of 4EC6F measured with red light (λ = 690 nm) across the N-N_X_ phase transition. The black line shows the derivative d(Δ*n*)/d*T*, and this clearly shows the transition temperature associated with the N_X_ phase. (b–d) Optical textures of N, N_X_, and N_F_ phases observed between crossed polarizers in a 1.8 μm-thick cell with planar anchoring, with the chevron defects and focal conic-like defects appearing in the N_X_ and N_F_ phases, respectively.

For the studied materials, the N-N_X_ phase transition is weakly first order. It is accompanied by only a small, step-like increase of optical birefringence of less than 0.001 for 4EC6F (Figure 4a), and this decreases on increasing the terminal alkyl chain length. Thus, one can assume that the orientational order of the molecules remains similar in the N and N_X_ phases.

**Figure 5:**
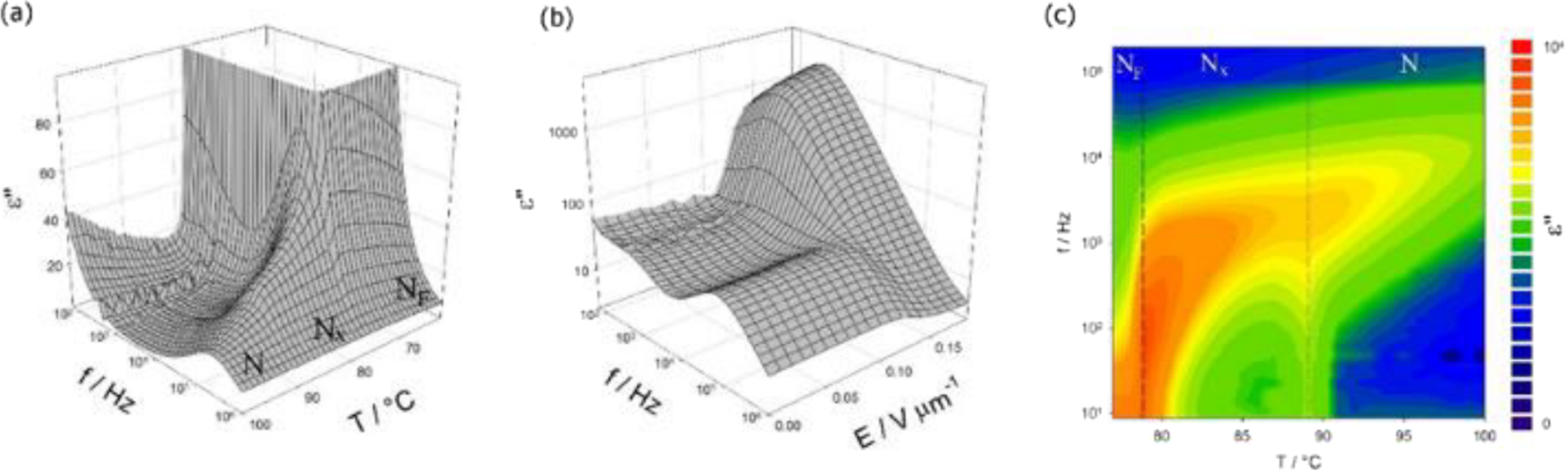
Imaginary part of dielectric permittivity measured for 4EC6F (*n* = 4): (a) temperature and frequency dependence across the N-N_X_-N_F_ phase sequence, (b) frequency and bias field dependence in the N_X_ phase (at *T* = 80 °C); where the high-frequency mode is suppressed and the lower-frequency ferroelectric mode is excited above the threshold field of 0.12 V/μm; and (c) map showing the evolution of the “ferroelectric” mode vs temperature and frequency. The measurements were performed under a bias electric field of 0.3 V/μm.

In optical studies performed using planar aligned cells, there is clearly a transition detected at the temperature described by the birefringence measurements. In the N phase, there is a uniform texture observed, and on entering the N_X_ phase, the flickering characteristic to non-polar phases ceases and chevron-like defects appear a few degrees below the phase transition (Figure 4). The dielectric studies performed for homologue 4EC6F show a weak dielectric mode in the N phase (with a relaxation frequency ∼ 10^5^ Hz) that continuously slows down but increases in strength through the entire range of the N and N_X_ phases. The N-N_X_ phase transition is marked by only a slight change in the value of the mode strength (Figure 5a). This mode might be ascribed to the non-collective rotations of molecules with strong dipole moments around their highest inertia axis. Entering the N_F_ phase, there is a dramatic change in the dielectric response, and a very strong, low relaxation frequency (∼10^3^ Hz) mode appears. Furthermore, in optical studies, there is also a clear transition observed upon entry to the N_F_ phase with the emergence of a blocky type texture with some focal conic-like defects (Figure 4).^13^ In the N_X_ phase under a weak bias electric field, the relaxation mode due to non-collective molecular rotations is quenched, and the mode due to the collective ferroelectric fluctuation is excited. Suppressing the non-collective fluctuations by a bias electric field allows us to follow the evolution of the “ferroelectric” mode (∼10^3^ Hz) over the whole temperature range (Figure 5b,c). This mode slightly “softens” at the N-N_X_ phase transition as the polar order in this phase is weak, but the transition to the N_F_ phase is marked by a much stronger critical lowering of the mode frequency and an increase of its strength. Therefore, typical critical behavior is observed.

**Figure 6:**
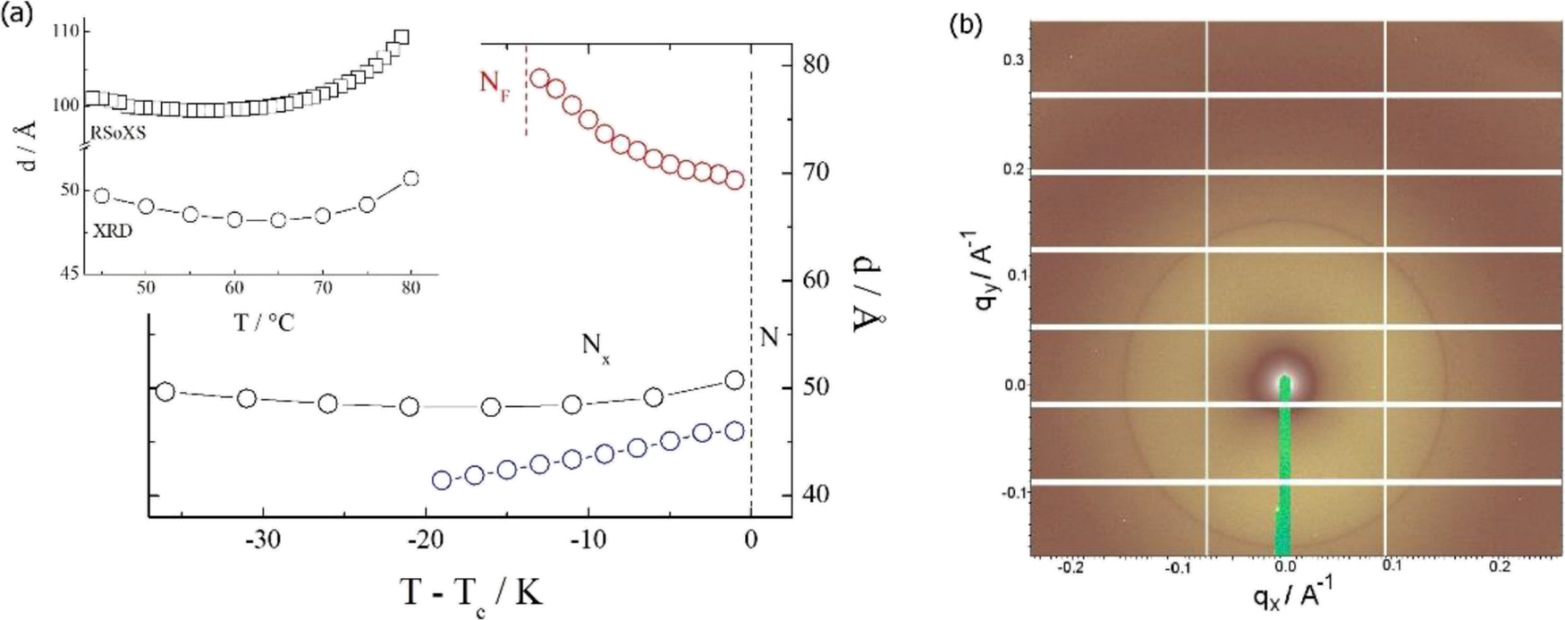
(a) Periodicity of the antiferroelectric domain structure in the N_X_ phase, *d*, vs temperature for the *n*EC6F series with *n* = 4 (red), *n* = 5 (black), and *n* = 6 (blue) measured using XRD. In the inset: comparison of the periodicities deduced from non-resonant (XRD) and resonant (RSoXS) studies for 5EC6F; (b) 2D XRD pattern registered for *n* = 6 in the N_X_ phase at *T* = 40 °C. The sharp signal at *q* = 0.15 Å^–1^ is due to the periodic structure of antiferroelectric domains, while the diffuse signal centered at *q* = 0.26 Å^–1^ reflects the short-range positional order of molecules along the director of the nematic phase.

In order to probe the structure of the N_X_ phase, small-angle XRD studies (SAXS) were performed. Using a strong synchrotron source, in addition to the diffuse signal due to the short-range positional order of the molecules, which is typical for the nematic phase, there was also a separate sharp, machine resolution limited signal (Figure 6). This observation proved that there was long-range ordering within the phase due to the periodic structure of the antiferroelectric domains. The low intensity of the signal shows that the related electron density modulation is very weak. The signal position depends on the terminal alkyl chain such that higher values of *n* give shorter periodicities, being deep within the N_X_ phase, around 75, 50, and 40 Å (just 20–10 molecular widths) for 4EC6F, 5EC6F, and 6EC6F, respectively. It would appear that the size of the domains defining the N_X_ structure can be correlated with the tendency to form the N_F_ phase. The size of the domains increases on approaching the transition to the N_F_ phase, and this tendency is clearly seen for 4EC6F. However, for 5EC6F, a much weaker and non-monotonic temperature dependence is observed. For 6EC6F, which is the homologue with the longest terminal alkyl chain length, the size of the domains monotonically decreases on cooling, suggesting that for this compound the tendency to form the ferroelectric nematic phase is very weak and therefore does not influence the domain size.

For 5EC6F, resonant soft X-ray scattering studies (RSoXS) were also conducted. The diffraction signal under the resonance condition is sensitive to the orientation of the molecules unlike conventional XRD. The RSoXS signal was found at a doubled periodicity that was detected in the SAXS measurements (Figure 6), and this clearly confirms that the structure is related to an antiparallel orientation of molecules in neighboring domains.

**Figure 7:**
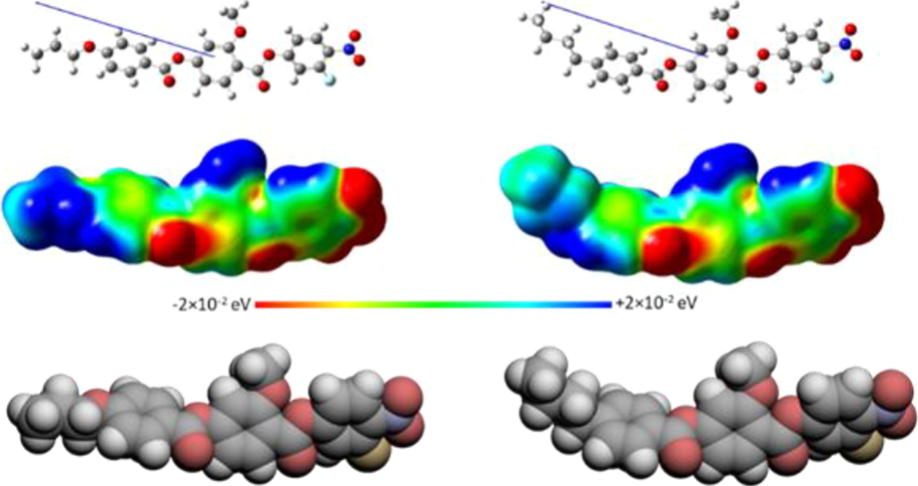
Molecular modeling of (left) 3OEC3F and (right) 4EC6F calculated at the B3LYP/6-31(d) level of theory. The molecules are visualized using (top) ball and stick models, (middle) electrostatic potential surfaces, and (bottom) space-filling models. The arrow indicates the direction of the calculated dipole moment, with the head representing positive charge moving to the base which is negative.

For comparison, we studied the homologous series *n*OEC3F, in which the terminal alkyl chain is replaced by an alkyloxy chain. Although in general the stability of the liquid crystalline phases is increased by introducing an oxygen atom between a terminal alkyl chain and the mesogenic unit, the tendency to form the polar N_F_ phase was diminished (Figure S1). This may be somewhat surprising considering that the average overall molecular dipole of the *n*OEC3F series is 13.0 D compared to 12.0 D for the *n*EC6F series (Figure 7). Apparently, larger longitudinal dipole moments are not exclusively the driving force for the formation of the N_F_ phase. Within the framework of the model of the N_F_ phase proposed by Madhusudana, the molecules are described by longitudinal surface charge density waves which interact to prevent the formation of antiparallel structures.^42^ In order to stabilize the ferroelectric nematic phase and promote the parallel alignment of the calamitic molecules, the amplitude of the charge density waves at either end of the molecule should be reduced. We have reported previously that this may be achieved using a fluorine atom at the *ortho* position to the terminal nitro group, rather than a hydrogen atom, in order to reduce the electron density associated with the nitro group.^30,31^ In this case, however, we are instead changing the electron density associated with the ring to which the terminal alkyl or alkyloxy chain is attached. An alkyloxy chain is a stronger activating functional group compared to an alkyl chain due to its enhanced electron-donating character, which means that there is a greater electron density in the terminal ring of the *n*OEC3F series compared to that of the *n*EC6F series (Figure 7).^43^ This increase in electron density will cause the amplitude of the surface charge density wave to increase for the *n*OEC3F series, and hence, the temperatures of the N-N_F_ and N-N_X_ phase transitions show a more pronounced decrease on the elongation of terminal chain for the alkyloxy derivatives when compared to their alkyl counterparts. This decrease may also be attributed, to some extent, to shape effects, given that the alkyloxy chain lies more or less in the plane of the mesogenic unit to which it is attached, whereas the alkyl chain protrudes at an angle, and this will also disrupt the anti-parallel correlations between the molecules (Figure 7). This weaker tendency to form ferroelectric phases in the *n*OEC3F series was also confirmed by XRD studies performed for the material 3OEC3F. The periodicity of the antiferroelectric domain structure in the N_X_ phase in the temperature range of 65–35 °C varied from 45 to 42 Å (Figure S2), which is less than that found for the alkyl terminated analogue 4EC6F, having the same total length of terminal chain. Measurements performed under the resonance condition, RSoXS, revealed doubled periodicity of the domain structure (Figure S2), as found for 5EC6F.

The dielectric measurements for 3OEC3F revealed two clear relaxation modes in the whole temperature range of the N_X_ and N phases (Figure S3). As described for 4EC6F, upon the application of a bias electric field in the N_X_ phase, the non-collective, higher frequency mode is quenched and instead the “ferroelectric mode” is excited. In 3OEC3F, the temperature evolution of this “ferroelectric mode” under a bias field showed a very weak softening behavior at the N-N_X_ phase transition, and this softening was much less pronounced than observed for the analogous compound 4EC6F. Such behavior is consistent with the optical observations, as there was a nearly smooth evolution of optical birefringence across the N–N_X_ phase transition (Figure S4), revealing its nearly continuous character and weak polar ordering in the domains.

## Conclusions

In conclusion, the results obtained indicate that the N_X_ phase is built from small polar regions, which form a regular antiferroelectric structure. The dielectric response measured indicates that the polar order in these regions is weak and that the phase transition to the conventional non-polar nematic is very weakly first order. The periodicity of the antiferroelectric domains array in the N_X_ phase increases with increasing ferroelectric interactions in the system, and the closer it is to the transition to the ferroelectric nematic phase, the wider these domains become. The question remains what causes the density modulation responsible for the weak X-ray signal in the N_X_ phase? It is possible that either the domain walls have slightly different densities than the ferroelectric domains or that the polarization splays or its magnitude is modulated across the domain, leading to slightly different densities at the domain boundaries.

## Supporting Information Available

Synthetic routes used to prepare the *n*EC6F and *n*OEC3F series; detailed description of the preparation of these compounds, including the structural characterization data for all intermediates and final products; and phase diagram for the nOEC3F series with supporting XRD, birefringence, and dielectric measurements (PDF)
