# Supplementary material for: To Be or Not To Be Polar: The Ferroelectric and Antiferroelectric Nematic Phases
Source: ACS Omega. 2023 Sep 18;8(39):36562–8. doi: 10.1021/acsomega.3c05884 (PMC10552116; doi:10.1021/acsomega.3c05884)
Supplement: Supplementary file 1 — ao3c05884_si_001.pdf [file ao3c05884_si_001.pdf]

To be or not to be polar: the ferroelectric and antiferroelectric nematic phases

†Ewan Cruickshank[a], Paulina Rybak[b], Magdalena M. Majewska[b], Shona Ramsay[a], Cheng Wang[c], Chenhui Zhu[c], Rebecca Walker[a], John M. D. Storey[a], Corrie T. Imrie[a], Ewa Gorecka[b], Damian Pociecha*[b]

[a] Department of Chemistry, School of Natural and Computing Sciences, University of Aberdeen, AB24 3UE, UK.

[b] University of Warsaw, Faculty of Chemistry, ul. Zwirki i Wigury 101, 02-089, Warsaw, Poland

[c] Advanced Light Source, Lawrence Berkeley National Laboratory, 1 Cyclotron Road, Berkeley, 94720 CA, USA.

†Present Address: School of Pharmacy and Life Sciences, Robert Gordon University, Aberdeen, AB10 7GJ, UK.

Experimental

Reagents

All reagents and solvents that were available commercially were purchased from Sigma Aldrich, Fisher Scientific or Fluorochem and were used without further purification unless otherwise stated.

Thin Layer Chromatography

Reactions were monitored using thin layer chromatography, and the appropriate solvent system, using aluminium-backed plates with a coating of Merck Kieselgel 60 F254 silica which were purchased from Merck KGaA. The spots on the plate were visualised by UV light (254 nm).

Column Chromatography

For normal phase column chromatography, the separations were carried out using silica gel grade 60 Å, 40-63 µm particle size, purchased from Fluorochem and using an appropriate solvent system.

Structure Characterisation

All final products and intermediates that were synthesised were characterised using ^{1}H NMR, ^{19}F NMR, ^{13}C NMR and infrared spectroscopies. The NMR spectra were recorded on a 400 MHz Bruker Avance III HD NMR spectrometer. The infrared spectra were recorded on a Perkin Elmer Spectrum Two FTIR with an ATR diamond cell.

Purity Analysis

In order to determine the purity of the final products, high-resolution mass spectrometry was carried out using a Waters XEVO G2 Q-Tof mass spectrometer by Dr. Morag Douglas at the University of Aberdeen.

Synthesis and Analytical Data

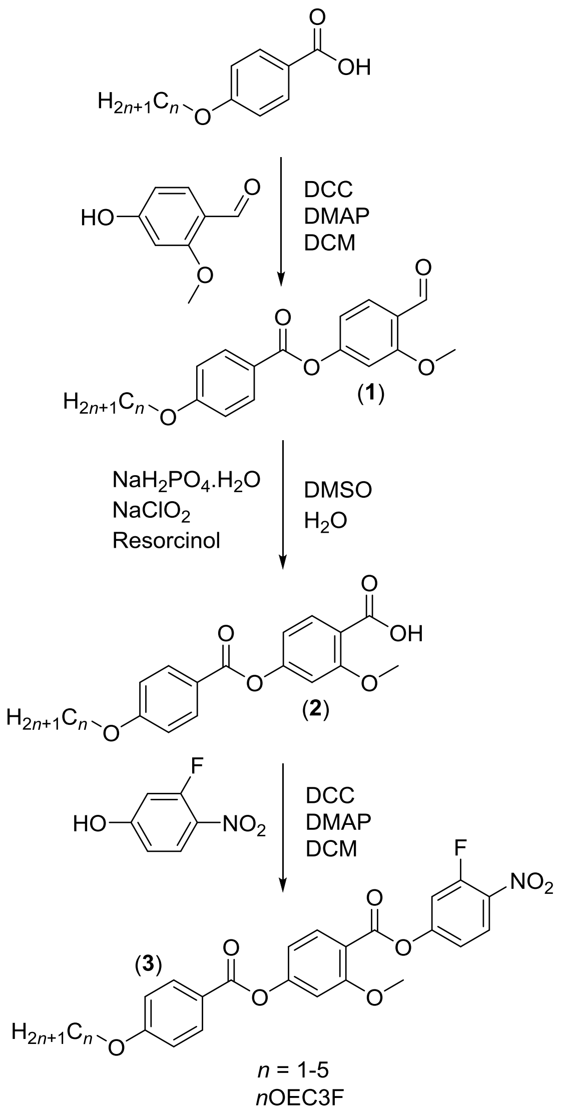

4-Formyl-3-methoxyphenyl 4-alkyloxybenzoates (1)

To a pre-dried flask flushed with argon, 4-alkyloxybenzoic acid of the appropriate chain length (1 eq), 4-hydroxy-2-methoxybenzaldehyde (1.1 eq) and 4-dimethylaminopyridine (0.13 eq) were added. The solids were solubilised with dichloromethane (100 mL) and tetrahydrofuran (20 mL) while being stirred for 10 min before *N,N′*-dicyclohexylcarbodiimide (1.3 eq) was added to the flask and the reaction was allowed to proceed overnight. The quantities of the reagents used in each reaction are listed in **Table S1**. The extent of the reaction was monitored by TLC using an appropriate solvent system (RF values quoted in the product data). The precipitate which formed was removed by vacuum filtration and the filtrate collected. The collected solvent was evaporated under vacuum to leave a solid which was recrystallised from hot ethanol (200 mL).

Table S1. Quantities of reagents used in the syntheses of the 4-formyl-3-methoxyphenyl 4-alkyloxybenzoates (**1**)

n	4-Alkyloxybenzoic Acid	4-Hydroxy-2-methoxybenzaldehyde	4-Dimethylaminopyridine	*N,N'*-Dicyclohexylcarbodiimide
1	3.00 g, 0.0197 mol	3.30 g, 0.0217 mol	0.313 g, $2.56{\times}10^{-3}$ mol	5.28 g, 0.0256 mol
2	3.00 g, 0.0181 mol	3.03 g, 0.0199 mol	0.287 g, $2.35{\times}10^{-3}$ mol	4.85 g, 0.0235 mol
3	3.00 g, 0.0166 mol	1.54 g, 0.0183 mol	0.264 g, $2.16{\times}10^{-3}$ mol	4.46 g, 0.0216 mol
4	3.00 g, 0.0150 mol	2.51 g, 0.0165 mol	0.238 g, $1.95{\times}10^{-3}$ mol	4.02 g, 0.0195 mol
5	3.00 g, 0.0144 mol	2.40 g, 0.0158 mol	0.228 g, $1.87{\times}10^{-3}$ mol	3.86 g, 0.0187 mol

1.1 4-Formyl-3-methoxyphenyl 4-methoxybenzoate

White solid. Yield: 4.54 g, 80.5 %. RF: 0.542 (40 % ethyl acetate:60 % 40:60 petroleum ether). M.P = 145 °C

v_{max}/cm^{-1}: 2844, 1735, 1678, 1602, 1589, 1581, 1511, 1496, 1475, 1456, 1416, 1401, 1315, 1249, 1194, 1171, 1154, 1118, 1101, 1054, 1024, 1006, 946, 878, 841, 821, 807, 785, 757, 734, 686, 666, 639, 624, 605, 562, 509, 501, 463, 436, 405

δ_H/ppm (400 MHz, $CDCl_3$): 10.40 (1 H, s, (C=O)-H), 8.12 (2 H, d, J 8.9 Hz, Ar-H), 7.87 (1 H, d, J 8.5 Hz, Ar-H), 6.97 (2 H, d, J 8.9 Hz, Ar-H), 6.88 (2 H, m, Ar-H), 3.91 (3 H, s, O-$\underline{CH_3}$), 3.88 (3 H, s, O-$\underline{CH_3}$)

δ_C/ppm (100 MHz, $CDCl_3$): 188.63, 164.24, 164.13, 162.84, 157.24, 132.42, 129.82, 122.55, 121.10, 114.36, 114.00, 105.82, 55.92, 55.57

1.2 4-Formyl-3-methoxyphenyl 4-ethoxybenzoate

White solid. Yield: 3.58 g, 65.9 %. RF: 0.781 (60 % ethyl acetate:40 % 40:60 petroleum ether). M.P = 134 °C

ν_{max}/cm^{-1}: 2979, 2897, 1728, 1680, 1604, 1589, 1514, 1489, 1466, 1450, 1423, 1414, 1387, 1254, 1190, 1121, 1097, 1068, 1037, 1025, 946, 921, 877, 853, 836, 807, 789, 758, 739, 690, 673, 626, 614, 563, 511, 500, 474, 461, 447, 423, 412, 405

δ_H/ppm (400 MHz, DMSO-d_6): 10.31 (1 H, s, (C=O)-H), 8.08 (1 H, d, J 8.7 Hz, Ar-H), 7.78 (1 H, d, J 8.4 Hz, Ar-H), 7.21 (1 H, d, J 1.9 Hz, Ar-H), 7.12 (1 H, d, J 8.7 Hz, Ar-H), 7.00 (1 H, dd, J 8.4 Hz, 1.9 Hz, Ar-H), 7.00 (1 H, d, J 2.3 Hz, Ar-H), 4.15 (2 H, quart, 7.0 Hz, O-$\underline{CH_2}$-CH_3), 3.91 (3 H, s, O-$\underline{CH_3}$), 1.36 (3 H, t, 7.0 Hz, O-CH_2-$\underline{CH_3}$)

δ_C/ppm (100 MHz, DMSO-d_6): 188.35, 163.75, 163.37, 162.72, 157.16, 132.33, 129.33, 122.12, 120.42, 114.83, 114.80, 107.13, 63.88, 56.52, 14.56

1.3 4-Formyl-3-methoxyphenyl 4-propoxybenzoate

Off-white solid. Yield: 2.17 g, 70.6 %. RF: 0.611 (40 % ethyl acetate:60 % 40:60 petroleum ether). M.P = 101 °C

ν_{max}/cm^{-1}: 2965, 2941, 2879, 1739, 1725, 1682, 1602, 1578, 1510, 1498, 1468, 1450, 1423, 1415, 1397, 1248, 1197, 1187, 1173, 1157, 1119, 1102, 1053, 1030, 1007, 948, 905, 875, 847, 840, 816, 796, 758, 737, 675, 645, 629, 615, 563, 505, 492, 466, 452, 415

δ_H/ppm (400 MHz, $CDCl_3$): 10.42 (1 H, s, (C=O)-H), 8.13 (2 H, d, J 8.9 Hz, Ar-H), 7.90 (1 H, d, J 9.0 Hz, Ar-H), 6.98 (2 H, d, J 8.9 Hz, Ar-H), 6.89 (2 H, m, Ar-H), 4.02 (2 H, t, J 6.6 Hz, O-$\underline{CH_2}$-CH_2-), 3.94 (3 H, s, O-$\underline{CH_3}$), 1.86 (2 H, m, O-CH_2-$\underline{CH_2}$-CH_3), 1.07 (3 H, t, J 7.4 Hz, O-CH_2-CH_2-$\underline{CH_3}$)

δ_C/ppm (100 MHz, $CDCl_3$): 188.83, 164.34, 164.01, 162.98, 157.40, 132.56, 130.04, 122.71, 120.98, 114.58, 114.52, 105.94, 70.00, 56.06, 22.58, 10.61

1.4 4-Formyl-3-methoxyphenyl 4-butoxybenzoate

White solid. Yield: 2.72 g, 55.2 %. RF: 0.279 (100 % dichloromethane). M.P = 89 °C

ν_{max}/cm^{-1}: 2969, 2951, 2909, 2871, 1733, 167, 1601, 1589, 1578, 1511, 1491, 1475, 1461, 1440, 1416, 1400, 1317, 1249, 1200, 1173, 1156, 1121, 1101, 1066, 1051, 1028, 1008, 974, 962, 946, 882, 849, 828, 811, 793, 758, 743, 730, 690, 673, 647, 628, 615, 564, 511, 499, 464, 431, 414

δ_H/ppm (400 MHz, $CDCl_3$): 10.42 (1 H, s, (C=O)-H), 8.13 (2 H, d, J 9.0 Hz, Ar-H), 7.90 (1 H, d, J 9.0 Hz, Ar-H), 6.98 (2 H, d, J 9.0 Hz, Ar-H), 6.89 (2 H, m, Ar-H), 4.06 (2 H, t, J 6.5 Hz, O-$\underline{CH_2}$-CH_2-), 3.94 (3 H, s, O-$\underline{CH_3}$), 1.81 (2 H, m, O-CH_2-$\underline{CH_2}$-CH_2-), 1.52 (2 H, m, O-CH_2-CH_2-$\underline{CH_2}$-CH_3), 1.00 (3 H, t, J 7.4 Hz, O-CH_2-CH_2-CH_2-$\underline{CH_3}$)

δ_C/ppm (100 MHz, $CDCl_3$): 188.69, 164.20, 163.89, 162.85, 157.26, 132.42, 129.90, 122.57, 120.83, 114.44, 114.38, 105.80, 68.09, 55.92, 31.12, 19.20, 13.83

1.5 4-Formyl-3-methoxyphenyl 4-pentyloxybenzoate

White solid. Yield: 2.98 g, 60.4 %. RF: 0.256 (100 % dichloromethane). M.P = 98 °C

ν_{max}/cm^{-1}: 2941, 2873, 1729, 1682, 1601, 1578, 1512, 1499, 1489, 1463, 1416, 1397, 1310, 1248, 1196, 1172, 1154, 1101, 1052, 1028, 1008, 945, 887, 876, 848, 841, 815, 796, 758, 737, 722, 689, 674, 646, 628, 615, 646, 628, 615, 564, 506, 480, 462, 427, 408

δ_H/ppm (400 MHz, $CDCl_3$): 10.42 (1 H, s, (C=O)-H), 8.13 (2 H, d, J 9.0 Hz, Ar-H), 7.90 (1 H, d, J 9.0 Hz, Ar-H), 6.98 (2 H, d, J 9.0 Hz, Ar-H), 6.89 (2 H, m, Ar-H), 4.05 (2 H, t, J 6.6 Hz, O-$\underline{CH_2}$-CH_2-), 3.94 (3 H, s, O-$\underline{CH_3}$), 1.83 (2 H, m, O-CH_2-$\underline{CH_2}$-CH_2-), 1.44 (4 H, m, O-CH_2-CH_2-$\underline{CH_2}$-$\underline{CH_2}$-CH_3), 0.95 (3 H, t, J 7.1 Hz, O-CH_2-CH_2-CH_2-CH_2-$\underline{CH_3}$)

δ_C/ppm (100 MHz, $CDCl_3$): 188.69, 164.20, 163.88, 162.85, 157.26, 132.42, 129.90, 122.58, 120.83, 114.44, 114.38, 105.80, 68.40, 55.92, 28.78, 28.13, 22.44, 14.02

4-((4-Alkyloxybenzoyl)oxy)-2-methoxybenzoic acid (2)

To a pre-dried flask flushed with argon, **Compound 1** (1 eq) and resorcinol (1.5 eq) were solubilised in DMSO (100 mL). Sodium chlorite (4 eq) and sodium hydrogen phosphate monohydrate (3.5 eq) were solubilised in water (60 mL) before being slowly poured into the reaction flask and the resultant mixture was stirred at room temperature overnight. The quantities of the reagents used in each reaction are listed in **Table S2**. The extent of the reaction was monitored by TLC using an appropriate solvent system (RF values quoted in the product data). The reaction mixture was diluted with water (200 mL) and the pH of the mixture was adjusted to 1 using 32% hydrochloric acid (≈ 30 mL). A white solid precipitated after acidification which was collected by vacuum filtration and recrystallised from hot ethanol (250 mL).

Table S2. Quantities of reagents used in the syntheses of the 4-((4-alkyloxybenzoyl)oxy)-2-methoxybenzoic acids (**2**)

n	(**1**)	Sodium Chlorite	Sodium Hydrogen Phosphate Monohydrate	Resorcinol
1	4.30 g, 0.0150 mol	5.43 g, 0.0600 mol	7.24 g, 0.0525 mol	2.48 g, 0.0225 mol
2	3.40 g, 0.0113 mol	4.09 g, 0.0452 mol	5.46 g, 0.0396 mol	1.87 g, 0.0170 mol
3	2.00 g, 6.36×10^{-3} mol	2.31 g, 0.0255 mol	3.08 g, 0.0223 mol	1.05 g, 9.54×10^{-3} mol
4	2.50 g, 7.61×10^{-3} mol	2.76 g, 0.0305 mol	3.67 g, 0.0266 mol	1.26 g, 0.0114 mol
5	2.80 g, 8.18×10^{-3} mol	2.96 g, 0.0327 mol	3.95 g, 0.0286 mol	1.35 g, 0.0123 mol

2.1 4-((4-Methoxybenzoyl)oxy)-2-methoxybenzoic acid

Yield: 3.95 g, 87.1 %. RF: 0.029 (40 % ethyl acetate:60 % 40:60 petroleum ether). M.P = 204 °C

ν_{max}/cm^{-1}: 3012, 1723, 1700, 1667, 1606, 1579, 1517, 1501, 1461, 1435, 1424, 1401, 1309, 1247, 1189, 1173, 1164, 1134, 1103, 1092, 1061, 1024, 952, 927, 876, 847, 826, 817, 799, 772, 760, 737, 692, 659, 626, 606, 553, 511, 457, 439

δ_H/ppm (400 MHz, DMSO-d_6): 12.65 (1 H, s, OH), 8.08 (2 H, d, J 8.8 Hz, Ar-H), 7.74 (1 H, d, J 8.4 Hz, Ar-H), 7.10 (3 H, m, Ar-H), 6.91 (1 H, dd, J 8.4 Hz, 1.9 Hz, Ar-H), 3.88 (3 H, s, O-$\underline{CH_3}$), 3.82 (3 H, s, O-$\underline{CH_3}$)

δ_C/ppm (100 MHz, DMSO-d_6): 167.05, 164.32, 164.27, 159.90, 154.86, 132.58, 132.31, 121.17, 119.04, 114.77, 114.09, 107.30, 56.58, 56.14

2.2 4-((4-Ethoxybenzoyl)oxy)-2-methoxybenzoic acid

Yield: 2.33 g, 65.2 %. RF: 0.020 (100 % dichloromethane). M.P = 202 °C

ν_{max}/cm^{-1}: 2983, 2944, 1735, 1694, 1666, 1604, 1577, 1509, 1476, 1459, 1433, 1398, 1328, 1301, 1242, 1188, 1161, 1132, 1120, 1101, 1090, 1056, 1026, 1004, 953, 924, 874 ,848 ,835, 825, 813, 800, 791, 772, 759, 738, 690, 670, 626, 595, 522, 510, 446, 435, 406

δ_H/ppm (400 MHz, DMSO-d_6): 12.59 (1 H, s, OH), 7.97 (2 H, d, J 8.7 Hz, Ar-H), 7.73 (1 H, d, J 8.4 Hz, Ar-H), 7.01 (1 H, d, J 2.0 Hz, Ar-H), 6.85 (1 H, dd, J 8.4 Hz, 2.0 Hz, Ar-H), 6.72 (1 H, d, J 2.2 Hz, Ar-H), 6.68 (1 H, dd, J 8.7 Hz, 2.2 Hz, Ar-H), 4.15 (2 H, quart, 7.0 Hz, O-$\underline{CH_2}$-CH_3), 3.91 (3 H, s, O-$\underline{CH_3}$), 1.36 (3 H, t, 7.0 Hz, O-CH_2-$\underline{CH_3}$)

δ_C/ppm (100 MHz, DMSO-d_6): 166.58, 163.80, 163.15, 159.43, 154.40, 132.12, 131.84, 120.52, 118.57, 114.67, 113.62, 106.84, 63.72, 56.12, 14.46

2.3 4-((4-Propoxybenzoyl)oxy)-2-methoxybenzoic acid

Yield: 1.62 g, 77.1 %. RF: 0.027 (100 % dichloromethane). M.P = 137 °C

ν_{max}/cm^{-1}: 2974, 2945, 2878, 1734, 1688, 1664, 1601, 1577, 1507, 1499, 1468, 1421, 1403, 1311, 1289, 1237, 1195, 1168, 1027, 1011, 945, 897, 883, 840, 790, 774, 757, 739, 691, 664, 627, 614, 595, 557, 512, 448, 419, 403

δ_H/ppm (400 MHz, DMSO-d_6): 12.65 (1 H, s, OH), 8.08 (2 H, d, J 8.7 Hz, Ar-H), 7.74 (1 H, d, J 8.4 Hz, Ar-H), 7.10 (3 H, m, Ar-H), 6.91 (1 H, dd, J 8.4 Hz, 1.8 Hz, Ar-H), 4.05 (2 H, t, J 6.6 Hz, O-$\underline{CH_2}$-CH_2-), 3.82 (3 H, s, O-$\underline{CH_3}$), 1.77 (2 H, m, O-CH_2-$\underline{CH_2}$-CH_3), 1.00 (3 H, t, J 7.4 Hz, O-CH_2-CH_2-$\underline{CH_3}$)

δ_C/ppm (100 MHz, DMSO-d_6): 166.58, 163.79, 163.32, 159.43, 154.40, 132.12, 131.83, 120.51, 118.57, 114.69, 113.62, 106.83, 69.46, 56.11, 21.88, 10.30

2.4 4-((4-Butoxybenzoyl)oxy)-2-methoxybenzoic acid

Yield: 2.30 g, 87.8 %. RF: 0.189 (100 % dichloromethane). M.P = 158 °C

ν_{max}/cm^{-1}: 2957, 2872, 1728, 1685, 1660, 1601, 1575, 1511, 1471, 1435, 1423, 1404, 1301, 1250, 1196, 1162, 1138, 1125, 1108, 1064, 1025, 1002, 952, 909, 871, 852, 840, 808, 789, 776, 758, 743, 690, 664, 643, 624, 596, 561, 514, 481, 467, 433, 407

δ_H/ppm (400 MHz, DMSO-d_6): 12.67 (1 H, s, OH), 8.07 (2 H, d, J 9.0 Hz, Ar-H), 7.74 (1 H, d, J 8.4 Hz, Ar-H), 7.11 (3 H, m, Ar-H), 6.91 (1 H, dd, J 8.4 Hz, 2.1 Hz, Ar-H), 4.09 (2 H, t, J 6.5 Hz, O-$\underline{CH_2}$-CH_2-), 3.81 (3 H, s, O-$\underline{CH_3}$), 1.73 (2 H, m, O-CH_2-$\underline{CH_2}$-CH_2-), 1.45 (2 H, m, O-CH_2-CH_2-$\underline{CH_2}$-CH_3), 0.94 (3 H, t, J 7.4 Hz, O-CH_2-CH_2-CH_2-$\underline{CH_3}$)

δ_C/ppm (100 MHz, DMSO-d_6): 166.65, 163.86, 163.37, 159.48, 154.45, 132.17, 131.91, 120.53, 118.58, 114.74, 113.68, 106.86, 67.77, 56.15, 30.59, 18.72, 13.74

2.5 4-((4-Pentyloxybenzoyl)oxy)-2-methoxybenzoic acid

Yield: 2.37 g, 80.8 %. RF: 0.184 (100 % dichloromethane). M.P = 142 °C

ν_{max}/cm^{-1}: 2945, 2871, 1725, 1685, 1663, 1599, 1575, 1510, 1466, 1433, 1421, 1404, 1300, 1243, 1194, 1159, 1138, 1107, 1096, 1065, 1028, 1007, 947, 874, 852, 842, 812, 778, 758, 740, 690, 664, 642, 625, 595, 559, 511, 466, 429

δ_H/ppm (400 MHz, DMSO-d_6): 12.67 (1 H, s, OH), 8.07 (2 H, d, J 8.8 Hz, Ar-H), 7.74 (1 H, d, J 8.4 Hz, Ar-H), 7.11 (3 H, m, Ar-H), 6.90 (1 H, dd, J 8.4 Hz, 2.1 Hz, Ar-H), 4.08 (2 H, t, J 6.5 Hz, O-$\underline{CH_2}$-CH_2-), 3.81 (3 H, s, O-$\underline{CH_3}$), 1.75 (2 H, m, O-CH_2-$\underline{CH_2}$-CH_2-), 1.38 (4 H, m, O-CH_2-CH_2-$\underline{CH_2}$-$\underline{CH_2}$-CH_3), 0.90 (3 H, t, J 7.0 Hz, O-CH_2-CH_2-CH_2-CH_2-$\underline{CH_3}$)

δ_C/ppm (100 MHz, DMSO-d_6): 166.65, 163.86, 163.36, 159.48, 154.45, 132.17, 131.91, 120.53, 118.57, 114.74, 113.67, 106.86, 68.05, 56.15, 28.24, 27.67, 21.93, 13.97

(3-Fluoro-4-nitrophenyl) 2-methoxy-4-((4-alkyloxybenzoyl)oxy)benzoates (3)

To a pre-dried flask flushed with argon, **Compound 2** (1 eq), 3-fluoro-4-nitrophenol (1.2 eq), and *N,N'*-dicyclohexylcarbodiimide (1.5 eq) were added to the flask. The solids were solubilised with dichloromethane (30 mL) and stirred for 30 min before 4-dimethylaminopyridine (0.15 eq) was added. The quantities of the reagents used in each reaction are listed in **Table S3**. The temperature of the reaction mixture was increased to room temperature and the reaction was allowed to proceed overnight. For the reactions with *N,N'*-dicyclohexylcarbodiimide, the white precipitate which formed was removed by vacuum filtration and the filtrate collected. The solvent was removed under vacuum and the crude product was purified using a silica gel column with an appropriate solvent system (RF values quoted in product data). The eluent fractions of interest were evaporated under vacuum to leave a white solid which was recrystallised from hot ethanol (60 mL).

Table S3. Quantities of reagents used in the syntheses of the (3-fluoro-4-nitrophenyl) 2-methoxy-4-((4-alkyloxybenzoyl)oxy)benzoates (**3**)

n	(**2**)	3-Fluoro-4-nitrophenol	4-Dimethylaminopyridine	*N,N'*-Dicyclohexylcarbodiimide
1	0.300 g, 9.92×10^{-4} mol	0.187 g, 1.19×10^{-3} mol	18 mg, 1.49×10^{-4} mol	0.307 g, 1.49×10^{-3} mol
2	0.300 g, 9.48×10^{-4} mol	0.177 g, 1.13×10^{-3} mol	17 mg, 1.42×10^{-4} mol	0.293 g, 1.42×10^{-3} mol
3	0.300 g, 9.08×10^{-4} mol	0.171 g, 1.09×10^{-3} mol	17 mg, 1.36×10^{-4} mol	0.281 g, 1.36×10^{-3} mol
4	0.300 g, 8.71×10^{-4} mol	0.164 g, 1.05×10^{-3} mol	16 mg, 1.31×10^{-4} mol	0.270 g, 1.31×10^{-3} mol
5	0.300 g, 8.37×10^{-4} mol	0.157 g, 1.00×10^{-3} mol	15 mg, 1.26×10^{-4} mol	0.260 g, 1.26×10^{-3} mol

3.1 (3-Fluoro-4-nitrophenyl) 2-methoxy-4-((4-methoxybenzoyl)oxy)benzoate (1OEC3F)

Yield: 0.285 g, 65.1 %. RF: 0.306 (100 % dichloromethane).

T_{CrI} 178 °C T_{N_FN} (138 °C) T_{NI} (154 °C)

ν_{max}/cm^{-1}: 1763, 1723, 1716, 1604, 1584, 1531, 1513, 1497, 1472, 1454, 1412, 1350, 1320, 1288, 1259, 1249, 1232, 1219, 1193, 1160, 1141, 1119, 1093, 1070, 1056, 1028, 1007, 989, 953, 877, 845, 820, 789, 760, 745, 680, 671, 624, 608, 568, 545, 524, 510, 423, 409

δ_H/ppm (400 MHz, DMSO-d$_6$): 8.30 (1 H, t, J 8.9 Hz, Ar-H), 8.11 (3 H, m, Ar-H), 7.73 (1 H, dd, J 12.1 Hz, 2.3 Hz, Ar-H), 7.43 (1 H, m, Ar-H), 7.26 (1 H, d, J 2.0 Hz, Ar-H), 7.15 (2 H, d, J 8.8 Hz, Ar-H), 7.07 (1 H, dd, J 8.6 Hz, 2.0 Hz, Ar-H), 3.90 (3 H, s, O-$\underline{CH_3}$), 3.89 (3 H, s, O-$\underline{CH_3}$)

δ_F/ppm (376 MHz, DMSO-d$_6$): -115.41

δ_C/ppm (100 MHz, DMSO-d$_6$): 164.42, 164.11, 162.14, 161.45, 157.05, 156.69, 156.16, 156.04, 154.43, 135.08, 135.01, 133.82, 132.67, 127.97, 127.95, 121.00, 119.75, 119.71, 115.00, 114.82, 114.61, 113.40, 113.16, 107.80, 56.98, 56.18

MS = $[M+Na]^+$: Calculated for $C_{22}H_{16}FNO_8Na$: 464.0758. Found: 464.0777. Difference: 4.1 ppm

3.2 (3-Fluoro-4-nitrophenyl) 2-methoxy-4-((4-ethoxybenzoyl)oxy)benzoate (2OEC3F)

Yield: 0.087 g, 20.2 %. RF: 0.410 (40 % ethyl acetate:60 % 40:60 petroleum ether).

T_{CrI} 168 °C $T_{N_FN_X}$ (103 °C) T_{N_XN} (118 °C) T_{NI} (153 °C)

ν_{max}/cm^{-1}: 1763, 1723, 1716, 1604, 1584, 1531, 1513, 1497, 1472, 1454, 1412, 1350, 1320, 1288, 1259, 1249, 1232, 1219, 1193, 1160, 1141, 1119, 1093, 1070, 1056, 1028, 1007, 989, 953, 877, 845, 820, 789, 760, 745, 680, 671, 624, 608, 568, 545, 524, 510, 423, 409

δ_H/ppm (400 MHz, $CDCl_3$): 8.15 (4 H, m, Ar-H), 7.28 (1 H, dd, J 11.4 Hz, 2.4 Hz, Ar-H), 7.21 (1 H, m, Ar-H), 6.98 (4 H, m, Ar-H), 4.15 (2 H, quart, 7.0 Hz, O-$\underline{CH_2}$-CH_3), 3.96 (3 H, s, O-$\underline{CH_3}$), 1.48 (3 H, t, 7.0 Hz, O-CH_2-$\underline{CH_3}$)

δ_F/ppm (376 MHz, $CDCl_3$): -113.38

δ_C/ppm (100 MHz, $CDCl_3$): 164.15, 163.75, 161.88, 161.81, 157.54, 156.86, 155.99, 155.89, 154.89, 134.69, 134.62, 133.78, 132.47, 127.12, 127.10, 120.79, 118.22, 118.18, 114.46, 114.44, 113.94, 112.56, 112.33, 106.50, 63.93, 56.35, 14.67

MS = $[M+H]^+$: Calculated for $C_{23}H_{19}FNO_8$: 456.1095. Found: 456.1100. Difference: 1.1 ppm

3.3 (3-Fluoro-4-nitrophenyl) 2-methoxy-4-((4-propoxybenzoyl)oxy)benzoate (3OEC3F)

Yield: 0.240 g, 56.3 %. RF: 0.361 (100 % dichloromethane).

T_{CrI} 145 °C T_{N_XN} (79 °C) T_{NI} (133 °C)

ν_{max}/cm^{-1}: 3077, 2964, 2881, 1752, 1722, 1601, 1576, 1526, 1508, 1490, 1482, 1423, 1408, 1343, 1283, 1252, 1216, 1190, 1158, 1143, 1129, 1092, 1066, 1042, 1006, 948, 889, 842, 806, 762, 747, 684, 670, 613, 601, 553, 525, 513, 457, 429, 419, 401

δ_H/ppm (400 MHz, $CDCl_3$): 8.15 (4 H, m, Ar-H), 7.28 (1 H, dd, J 11.4 Hz, 2.4 Hz, Ar-H), 7.21 (1 H, m, Ar-H), 6.97 (4 H, m, Ar-H), 4.03 (2 H, t, J 6.6 Hz, O-$\underline{CH_2}$-CH_2-), 3.96 (3 H, s, O-$\underline{CH_3}$), 1.87 (2 H, m, O-CH_2-$\underline{CH_2}$-CH_3), 1.07 (3 H, t, J 7.4 Hz, O-CH_2-CH_2-$\underline{CH_3}$)

δ_F/ppm (376 MHz, $CDCl_3$): -113.35

δ_C/ppm (100 MHz, $CDCl_3$): 163.99, 163.75, 161.67, 161.63, 157.34, 156.67, 155.79, 155.69, 154.69, 134.47, 134.40, 133.60, 132.27, 126.94, 126.92, 120.49, 118.05, 118.01, 114.28, 114.18, 113.76, 112.39, 112.15, 106.30, 69.68, 56.16, 22.26, 10.30

MS = $[M+H]^+$: Calculated for $C_{24}H_{21}FNO_8$: 470.1251. Found: 470.1268. Difference: 3.6 ppm

3.4 (3-Fluoro-4-nitrophenyl) 2-methoxy-4-((4-butoxybenzoyl)oxy)benzoate (4OEC3F)

Yield: 0.242 g, 55.1 %. RF: 0.351 (100 % dichloromethane).

T_{CrN} 128 °C T_{N_XN} (38 °C) T_{NI} 130 °C

ν_{max}/cm^{-1}: 2961, 2876, 1726, 1715, 1602, 1577, 1530, 1511, 1493, 1474, 1415, 1348, 1318, 1260, 1227, 1189, 1163, 1126, 1116, 1092, 1073, 1047, 1021, 968, 947, 885, 842, 820, 760, 744, 691, 672, 629, 616, 573, 546, 511, 461, 420, 403

δ_H/ppm (400 MHz, $CDCl_3$): 8.14 (4 H, m, Ar-H), 7.28 (1 H, dd, J 11.4 Hz, 2.4 Hz, Ar-H), 7.21 (1 H, m, Ar-H), 6.97 (4 H, m, Ar-H), 4.07 (2 H, t, J 6.5 Hz, O-$\underline{CH_2}$-CH_2-), 3.96 (3 H, s, O-$\underline{CH_3}$), 1.82 (2 H, m, O-CH_2-$\underline{CH_2}$-CH_2-), 1.53 (2 H, m, O-CH_2-CH_2-$\underline{CH_2}$-CH_3), 1.00 (3 H, t, J 7.4 Hz, O-CH_2-CH_2-CH_2-$\underline{CH_3}$)

δ_F/ppm (376 MHz, $CDCl_3$): -113.35

δ_C/ppm (100 MHz, $CDCl_3$): 164.30, 164.08, 161.98, 161.94, 157.66, 156.98, 156.11, 156.00, 155.01, 134.78, 134.71, 133.91, 132.58, 127.26, 127.24, 120.80, 118.36, 118.32, 114.59, 114.49, 114.07, 112.70, 112.47, 106.61, 68.23, 56.48, 31.24, 19.33, 13.97

MS = $[M+H]^+$: Calculated for $C_{25}H_{23}FNO_8$: 484.1408. Found: 484.1418. Difference: 2.1 ppm

3.5 (3-Fluoro-4-nitrophenyl) 2-methoxy-4-((4-pentyloxybenzoyl)oxy)benzoate (5OEC3F)

Yield: 0.176 g, 42.3 %. RF: 0.417 (100 % dichloromethane).

T_{CrN} 92 °C T_{NI} 118 °C

ν_{max}/cm^{-1}: 2935, 2871, 1761, 1718, 1601, 1578, 1529, 1510, 1486, 1474, 1414, 1346, 1318, 1259, 1223, 1192, 1152, 1124, 1115, 1093, 1079, 1043, 1023, 1005, 969, 886, 841, 818, 759, 744, 691, 672, 628, 614, 573, 545, 513, 462, 427, 415

δ_H/ppm (400 MHz, $CDCl_3$): 8.15 (4 H, m, Ar-H), 7.28 (1 H, dd, J 11.3 Hz, 2.4 Hz, Ar-H), 7.21 (1 H, m, Ar-H), 6.97 (4 H, m, Ar-H), 4.06 (2 H, t, J 6.6 Hz, O-$\underline{CH_2}$-CH_2-), 3.96 (3 H, s, O-$\underline{CH_3}$), 1.83 (2 H, m, O-CH_2-$\underline{CH_2}$-CH_2-), 1.44 (4 H, m, O-CH_2-CH_2-$\underline{CH_2\text{-}CH_2}$-$CH_3$), 0.95 (3 H, t, J 7.1 Hz, O-CH_2-CH_2-CH_2-CH_2-$\underline{CH_3}$)

δ_F/ppm (376 MHz, $CDCl_3$): -113.37

δ_C/ppm (100 MHz, $CDCl_3$): 164.31, 164.08, 162.00, 161.95, 157.67, 156.99, 156.12, 156.01, 155.02, 134.80, 134.73, 133.92, 132.59, 127.27, 127.24, 120.81, 118.37, 118.33, 114.60, 114.51, 114.08, 112.71, 112.47, 106.62, 68.55, 56.49, 28.91, 28.27, 22.58, 14.17

MS = $[M+H]^+$: Calculated for $C_{26}H_{25}FNO_8$: 498.1564. Found: 498.1582. Difference: 3.6 ppm

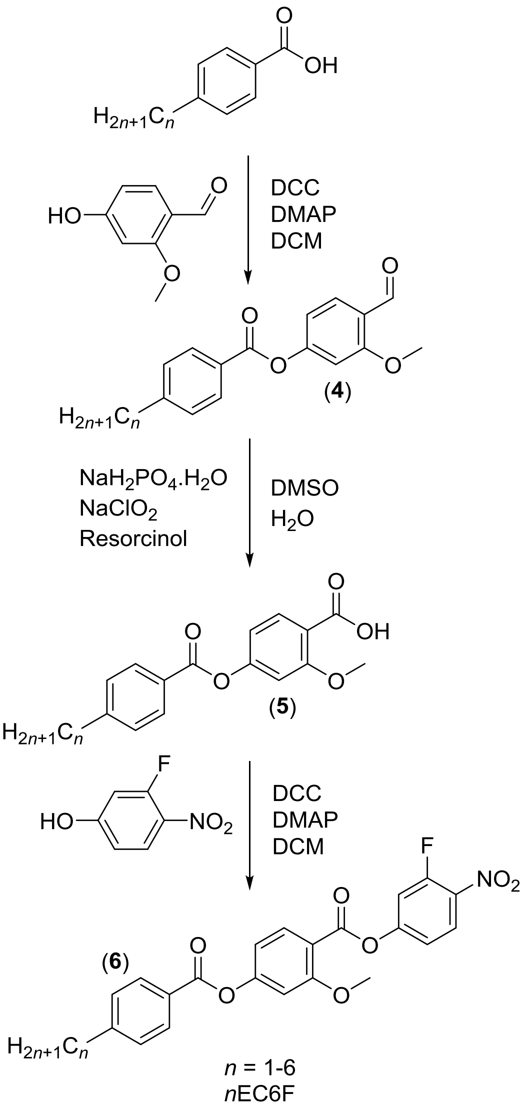

4-Formyl-3-methoxyphenyl 4-alkylbenzoates (4)

To a pre-dried flask flushed with argon, 4-alkylbenzoic acid of the appropriate chain length (1 eq), 4-hydroxy-2-methoxybenzaldehyde (1.1 eq) and 4-dimethylaminopyridine (0.13 eq) were added. The solids were solubilised with dichloromethane (100 mL) and tetrahydrofuran (20 mL) while being stirred for 10 min before *N*,*N'*-dicyclohexylcarbodiimide (1.3 eq) was added to the flask and the reaction was allowed to proceed overnight. The quantities of the reagents used in each reaction are listed in **Table S4**. The extent of the reaction was monitored by TLC using an appropriate solvent system (RF values quoted in the product data). The precipitate which formed was removed by vacuum filtration and the filtrate collected. The collected solvent was evaporated under vacuum to leave a solid which was recrystallised from hot ethanol (150 mL).

Table S4. Quantities of reagents used in the syntheses of the 4-formyl-3-methoxyphenyl 4-alkylbenzoates (**4**)

n	4-Alkylbenzoic Acid	4-Hydroxy-2-methoxybenzaldehyde	4-Dimethylaminopyridine	*N,N′*-Dicyclohexylcarbodiimide
1	2.50 g, 0.0184 mol	3.07 g, 0.0202 mol	0.292 g, 2.39×10^{-3} mol	4.93 g, 0.0256 mol
2	3.00 g, 0.0200 mol	3.34 g, 0.0220 mol	0.318 g, 2.60×10^{-3} mol	5.36 g, 0.0260 mol
3	3.00 g, 0.0183 mol	3.06 g, 0.0201 mol	0.291 g, 2.38×10^{-3} mol	4.91 g, 0.0238 mol
4	3.00 g, 0.0168 mol	2.81 g, 0.0185 mol	0.266 g, 2.18×10^{-3} mol	4.50 g, 0.0218 mol
5	3.00 g, 0.0156 mol	2.62 g, 0.0172 mol	0.248 g, 2.03×10^{-3} mol	4.19 g, 0.0203 mol
6	3.00 g, 0.0145 mol	2.43 g, 0.0160 mol	0.231 g, 1.89×10^{-3} mol	3.90 g, 0.0189 mol

4.1 4-Formyl-3-methoxyphenyl 4-methylbenzoate

White solid. Yield: 3.35 g, 67.4 %. RF: 0.550 (40 % ethyl acetate:60 % 40:60 petroleum ether). M.P = 117 °C

ν_{max}/cm^{-1}: 2869, 1739, 1679, 1599, 1587, 1493, 1474, 1465, 1417, 1403, 1379, 1262, 1248, 1200, 1182, 1157, 1120, 1099, 1063, 1027, 1017, 945, 872, 838, 822, 806, 788, 743, 686, 668, 627, 604, 561, 502, 467, 435, 409

δ_H/ppm (400 MHz, $CDCl_3$): 10.42 (1 H, s, (C=O)-H), 8.08 (2 H, d, J 8.3 Hz, Ar-H), 7.91 (1 H, d, J 7.9 Hz, Ar-H), 7.33 (2 H, d, J 8.3 Hz, Ar-H), 6.90 (2 H, m, Ar-H), 3.94 (3 H, s, O-$\underline{CH_3}$), 2.46 (3 H, s, Ar-$\underline{CH_3}$)

δ_C/ppm (100 MHz, $CDCl_3$): 188.80, 164.64, 162.98, 157.27, 145.13, 130.44, 130.06, 129.58, 126.29, 122.79, 114.47, 105.90, 56.06, 21.95

4.2 4-Formyl-3-methoxyphenyl 4-ethylbenzoate

White solid. Yield: 2.99 g, 52.6 %. RF: 0.600 (40 % ethyl acetate:60 % 40:60 petroleum ether). M.P = 105 °C

ν_{max}/cm^{-1}: 2971, 2880, 1732, 1680, 1600, 1583, 1488, 1470, 1457, 1416, 1399, 1305, 1252, 1191, 1179, 1151, 1100, 1065, 1051, 1042, 1024, 943, 888, 851, 816, 803, 777, 728, 695, 663, 636, 627, 599, 563, 497, 465, 455, 434, 422

δ_H/ppm (400 MHz, $CDCl_3$): 10.43 (1 H, s, (C=O)-H), 8.11 (2 H, d, J 8.2 Hz, Ar-H), 7.91 (1 H, d, J 8.9 Hz, Ar-H), 7.35 (2 H, d, J 8.2 Hz, Ar-H), 6.90 (2 H, m, Ar-H), 3.94 (3 H, s, O-$\underline{CH_3}$), 2.76 (2 H, quart, 7.6 Hz, Ar-$\underline{CH_2}$-CH_3), 1.29 (3 H, t, 7.6 Hz, Ar-CH_2-$\underline{CH_3}$)

δ_C/ppm (100 MHz, $CDCl_3$): 188.79, 164.64, 162.98, 157.28, 151.29, 130.56, 130.06, 128.40, 126.48, 122.79, 114.47, 105.90, 56.06, 29.22, 15.34

4.3 4-Formyl-3-methoxyphenyl 4-propylbenzoate

Off-white solid. Yield: 4.28 g, 78.4 %. RF: 0.583 (40 % ethyl acetate:60 % 40:60 petroleum ether). M.P = 86 °C

ν_{max}/cm^{-1}: 2955, 2928, 2859, 1739, 1680, 1599, 1582, 1487, 1464, 1415, 1393, 1311, 1270, 1239, 1193, 1174, 1149, 1100, 1072, 1054, 1015, 941, 901, 887, 851, 810, 793, 755, 737, 696, 670, 643, 634, 610, 560, 514, 500, 464, 449, 418, 406

δ_H/ppm (400 MHz, $CDCl_3$): 10.42 (1 H, s, (C=O)-H), 8.10 (2 H, d, J 8.2 Hz, Ar-H), 7.91 (1 H, d, J 9.0 Hz, Ar-H), 7.33 (2 H, d, J 8.2 Hz, Ar-H), 6.90 (2 H, m, Ar-H), 3.94 (3 H, s, O-$\underline{CH_3}$), 2.69 (2 H, t, J 7.4 Hz, Ar-$\underline{CH_2}$-CH_2-), 1.70 (2 H, m, Ar-CH_2-$\underline{CH_2}$-CH_3), 0.97 (3 H, t, J 7.4 Hz, Ar-CH_2-CH_2-$\underline{CH_3}$)

δ_C/ppm (100 MHz, $CDCl_3$): 188.81, 164.66, 162.98, 157.29, 149.79, 130.46, 130.06, 128.99, 126.50, 122.78, 114.47, 105.90, 56.06, 38.27, 24.36, 13.88

4.4 4-Formyl-3-methoxyphenyl 4-butylbenzoate

Off-white solid. Yield: 2.64 g, 50.4 %. RF: 0.342 (100 % dichloromethane). M.P = 61 °C

ν_{max}/cm^{-1}: 2951, 2929, 2868, 1739, 1673, 1603, 1580, 1486, 1470, 1448, 1414, 1404, 1373, 1308, 1273, 120, 1239, 1207, 1171, 1150, 1106, 1075, 1056, 1028, 1017, 944, 902, 870, 841, 804, 753, 731, 693, 657, 635, 621, 570, 520, 489, 46, 410, 403

δ_H/ppm (400 MHz, $CDCl_3$): 10.42 (1 H, s, (C=O)-H), 8.10 (2 H, d, J 8.4 Hz, Ar-H), 7.91 (1 H, d, J 8.9 Hz, Ar-H), 7.33 (2 H, d, J 8.4 Hz, Ar-H), 6.90 (2 H, m, Ar-H), 3.94 (3 H, s, O-$\underline{CH_3}$), 2.71 (2 H, t, J 7.6 Hz, Ar-$\underline{CH_2}$-CH_2-), 1.64 (2 H, m, Ar-CH_2-$\underline{CH_2}$-CH_2-), 1.38 (2 H, m, Ar-CH_2-CH_2-$\underline{CH_2}$-CH_3), 0.95 (3 H, t, J 7.3 Hz, Ar-CH_2-CH_2-CH_2-$\underline{CH_3}$)

δ_C/ppm (100 MHz, $CDCl_3$): 188.84, 164.66, 162.97, 157.28, 150.06, 130.47, 130.06, 128.94, 126.41, 122.74, 114.47, 105.89, 56.05, 35.95, 33.38, 22.45, 14.06

4.5 4-Formyl-3-methoxyphenyl 4-pentylbenzoate

Off-white solid. Yield: 2.22 g, 43.6 %. RF: 0.367 (100 % dichloromethane). M.P = 73 °C

ν_{max}/cm^{-1}: 2955, 2928, 2859, 2780, 1738, 1672, 1602, 1487, 1469, 1449, 1416, 1405, 1308, 1273, 1251, 1206, 1171, 1103, 1077, 1058, 1027, 1016, 944, 891, 870, 840, 808, 752, 727, 694, 658, 634, 623, 611, 571, 528, 488, 467, 441, 432, 413

δ_H/ppm (400 MHz, $CDCl_3$): 10.42 (1 H, s, (C=O)-H), 8.10 (2 H, d, J 8.1 Hz, Ar-H), 7.91 (1 H, d, J 8.9 Hz, Ar-H), 7.33 (2 H, d, J 8.1 Hz, Ar-H), 6.90 (2 H, m, Ar-H), 3.94 (3 H, s, O-$\underline{CH_3}$), 2.70 (2 H, t, J 7.8 Hz, Ar-$\underline{CH_2}$-CH_2-), 1.67 (2 H, tt, J 7.8 Hz, 7.2 Hz, Ar-CH_2-$\underline{CH_2}$-CH_2-), 1.34 (4 H, m, Ar-CH_2-CH_2-$\underline{CH_2}$-$\underline{CH_2}$-CH_3), 0.90 (3 H, t, J 7.0 Hz, Ar-CH_2-CH_2-CH_2-CH_2-$\underline{CH_3}$)

δ_C/ppm (100 MHz, $CDCl_3$): 188.83, 164.66, 162.97, 157.28, 150.08, 130.47, 130.05, 128.93, 126.40, 122.74, 114.46, 105.88, 56.04, 36.21, 31.54, 30.93, 22.63, 14.14

4.6 4-Formyl-3-methoxyphenyl 4-hexylbenzoate

Off-white solid. Yield: 2.52 g, 51.1 %. RF: 0.432 (100 % dichloromethane). M.P = 69 °C

ν_{max}/cm^{-1}: 2953, 2926, 2854, 2781, 1735, 1677, 1599, 1586, 1487, 1466, 1416, 1404, 1310, 1244, 1201, 1175, 1152, 1100, 1058, 1028, 1016, 945, 890, 841, 817, 796, 753, 726, 697, 669, 645, 634, 610, 564, 521, 496, 465, 408

δ_H/ppm (400 MHz, $CDCl_3$): 10.42 (1 H, s, (C=O)-H), 8.10 (2 H, d, J 8.3 Hz, Ar-H), 7.91 (1 H, d, J 9.0 Hz, Ar-H), 7.33 (2 H, d, J 8.3 Hz, Ar-H), 6.90 (2 H, m, Ar-H), 3.94 (3 H, s, O-$\underline{CH_3}$), 2.71 (2 H, t, J 7.6 Hz, Ar-$\underline{CH_2}$-CH_2-), 1.88 (2 H, tt, J 7.6 Hz, 6.9 Hz, Ar-CH_2-$\underline{CH_2}$-CH_2-), 1.32 (6 H, m, Ar-CH_2-CH_2-$\underline{CH_2}$-$\underline{CH_2}$-$\underline{CH_2}$-CH_3), 0.89 (3 H, t, J 6.9 Hz, Ar-CH_2-CH_2-CH_2-CH_2-CH_2-$\underline{CH_3}$)

δ_C/ppm (100 MHz, $CDCl_3$): 188.86, 164.67, 162.98, 157.29, 150.10, 130.48, 130.07, 128.94, 126.40, 122.74, 114.48, 105.89, 56.05, 36.26, 31.79, 31.22, 29.04, 22.72, 14.23

4-((4-Alkylbenzoyl)oxy)-2-methoxybenzoic acid (5)

To a pre-dried flask flushed with argon, **Compound 4** (1 eq) and resorcinol (1.5 eq) were solubilised in DMSO (100 mL or 120 mL for *n* = 3). Sodium chlorite (4 eq) and sodium hydrogen phosphate monohydrate (3.5 eq) were solubilised in water (60 mL or 80 mL for *n* = 3) before being slowly poured into the reaction flask and the resultant mixture was stirred at room temperature overnight. The quantities of the reagents used in each reaction are listed in **Table S5**. The extent of the reaction was monitored by TLC using an appropriate solvent system (RF values quoted in the product data). The reaction mixture was diluted with water (200 mL) and the pH of the mixture was adjusted to 1 using 32% hydrochloric acid (≈ 30 mL). A solid precipitated after acidification which was collected by vacuum filtration and recrystallised from hot ethanol (150 mL) or hot ethanol (70 mL) with 40:60 petroleum ether (60 mL) for *n* = 6.

Table S5. Quantities of reagents used in the syntheses of the 4-((4-alkyloxybenzoyl)oxy)-2-methoxybenzoic acids (**5**)

n	(**4**)	Sodium Chlorite	Sodium Hydrogen Phosphate Monohydrate	Resorcinol
1	3.10 g, 0.0115 mol	4.16 g, 0.0460 mol	5.56 g, 0.0403 mol	1.90 g, 0.0173 mol
2	2.80 g, 9.85×10^{-3} mol	3.56 g, 0.0394 mol	4.76 g, 0.0345 mol	1.63 g, 0.0148 mol
3	4.00 g, 0.0134 mol	4.85 g, 0.0536 mol	6.47 g, 0.0469 mol	2.21 g, 0.0201 mol
4	2.50 g, 8.00×10^{-3} mol	2.89 g, 0.0320 mol	3.86 g, 0.0280 mol	1.32 g, 0.0120 mol
5	2.10 g, 6.43×10^{-3} mol	2.32 g, 0.0257 mol	3.10 g, 0.0225 mol	1.06 g, 9.65×10^{-3} mol
6	2.40 g, 7.05×10^{-3} mol	2.55 g, 0.0282 mol	3.41 g, 0.0247 mol	1.17 g, 0.0106 mol

5.1 4-((4-Methylbenzoyl)oxy)-2-methoxybenzoic acid

Off-white solid. Yield: 2.54 g, 77.2 %. RF: 0.079 (40 % ethyl acetate:60 % 40:60 petroleum ether). M.P = 204 °C

ν_{max}/cm^{-1}: 2816, 1725, 1685, 1672, 1604, 1583, 1499, 1468, 1408, 1303, 1241, 1194, 1177, 1160, 1140, 1095, 1060, 1029, 1018, 947, 892, 838, 787, 770, 747, 668, 652, 593, 554, 481, 445

δ_H/ppm (400 MHz, DMSO-d_6): 12.67 (1 H, s, OH), 8.03 (2 H, d, J 8.0 Hz, Ar-H), 7.75 (1 H, d, J 8.4 Hz, Ar-H), 7.42 (2 H, d, J 8.0 Hz, Ar-H), 7.11 (1 H, d, J 2.0 Hz, Ar-H), 6.92 (1 H, dd, J 8.4 Hz, 2.0 Hz, Ar-H), 3.82 (3 H, s, O-$\underline{CH_3}$), 2.43 (3 H, s, Ar-$\underline{CH_3}$)

δ_C/ppm (100 MHz, DMSO-d_6): 166.58, 164.16, 159.45, 154.32, 144.72, 131.87, 129.93, 129.56, 125.93, 118.70, 113.58, 106.82, 56.13, 21.28

5.2 4-((4-Ethylbenzoyl)oxy)-2-methoxybenzoic acid

White solid. Yield: 1.73 g, 58.5 %. RF: 0.105 (40 % ethyl acetate:60 % 40:60 petroleum ether). M.P = 170 °C

ν_{max}/cm^{-1}: 2979, 2941, 1725, 1683, 1669, 1604, 1580, 1496, 1464, 1433, 1404, 1285, 1238, 1190, 1177, 1157, 1138, 1093, 1065, 1051, 1026, 1016, 979, 947, 889, 850, 781, 766, 734, 696, 650, 627, 594, 555, 496, 442, 418

δ_H/ppm (400 MHz, DMSO-d_6): 12.67 (1 H, s, OH), 8.06 (2 H, d, J 8.1 Hz, Ar-H), 7.75 (1 H, d, J 8.4 Hz, Ar-H), 7.45 (2 H, d, J 8.0 Hz, Ar-H), 7.11 (1 H, d, J 2.0 Hz, Ar-H), 6.92 (1 H, dd, J 8.4 Hz, 2.0 Hz, Ar-H), 3.82 (3 H, s, O-$\underline{CH_3}$), 2.73 (2 H, quart, 7.6 Hz, Ar-$\underline{CH_2}$-CH_3), 1.22 (3 H, t, 7.6 Hz, Ar-CH_2-$\underline{CH_3}$)

δ_C/ppm (100 MHz, DMSO-d_6): 166.58, 164.16, 159.44, 154.32, 150.75, 131.87, 130.05, 128.39, 126.17, 118.70, 113.59, 106.82, 56.13, 28.27, 15.18

5.3 4-((4-Propylbenzoyl)oxy)-2-methoxybenzoic acid

White solid. Yield: 2.58 g, 61.3 %. RF: 0.054 (40 % ethyl acetate:60 % 40:60 petroleum ether). M.P = 166 °C

ν_{max}/cm^{-1}: 2961, 2871, 1731, 1690, 1666, 1600, 1581, 1496, 1468, 1406, 1297, 1237, 1194, 1177, 1163, 1130, 1091, 1056, 1028, 1016, 940, 891, 851, 840, 788, 774, 758, 731, 692, 664, 634, 616, 594, 553, 491, 462, 441, 416

δ_H/ppm (400 MHz, DMSO-d_6): 12.66 (1 H, s, OH), 8.06 (2 H, d, J 8.2 Hz, Ar-H), 7.75 (1 H, d, J 8.4 Hz, Ar-H), 7.43 (2 H, d, J 8.2 Hz, Ar-H), 7.11 (1 H, d, J 2.0 Hz, Ar-H), 6.92 (1 H, dd, J 8.4 Hz, 2.0 Hz, Ar-H), 3.82 (3 H, s, O-$\underline{CH_3}$), 2.68 (2 H, t, J 7.6 Hz, Ar-$\underline{CH_2}$-CH_2-), 1.64 (2 H, m, Ar-CH_2-$\underline{CH_2}$-CH_3), 0.90 (3 H, t, J 7.3 Hz, Ar-CH_2-CH_2-$\underline{CH_3}$)

δ_C/ppm (100 MHz, DMSO-d_6): 166.57, 164.15, 159.44, 154.33, 149.14, 131.86, 129.95, 128.97, 126.21, 118.69, 113.59, 106.82, 56.12, 37.17, 23.73, 13.52

5.4 4-((4-Butylbenzoyl)oxy)-2-methoxybenzoic acid

White solid. Yield: 1.13 g, 43.0 %. RF: 0.162 (100 % dichloromethane). M.P = 115 °C

ν_{max}/cm^{-1}: 2955, 2932, 2872, 1727, 1699, 1677, 1603, 1582, 1495, 1466, 1453, 1429, 1406, 1245, 1190, 1177, 1158, 1131, 1103, 1093, 1061, 1028, 1015, 948, 934, 889, 847, 786, 772, 750, 725, 687, 664, 635, 610, 592, 554, 502, 468, 439, 416, 404

δ_H/ppm (400 MHz, DMSO-d_6): 12.68 (1 H, s, OH), 8.05 (2 H, d, J 8.3 Hz, Ar-H), 7.75 (1 H, d, J 8.4 Hz, Ar-H), 7.43 (2 H, d, J 8.3 Hz, Ar-H), 7.11 (1 H, d, J 2.1 Hz, Ar-H), 6.92 (1 H, dd, J 8.4 Hz, 2.1 Hz, Ar-H), 3.81 (3 H, s, O-$\underline{CH_3}$), 2.70 (2 H, t, J 7.6 Hz, Ar-$\underline{CH_2}$-CH_2-), 1.59 (2 H, m, Ar-CH_2-$\underline{CH_2}$-CH_2-), 1.31 (2 H, m, Ar-CH_2-CH_2-$\underline{CH_2}$-CH_3), 0.90 (3 H, t, J 7.3 Hz, Ar-CH_2-CH_2-CH_2-$\underline{CH_3}$)

δ_C/ppm (100 MHz, DMSO-d_6): 166.65, 164.21, 159.49, 154.38, 149.44, 131.93, 130.03, 128.97, 126.19, 118.70, 113.65, 106.86, 56.15, 34.88, 32.78, 21.74, 13.80

5.5 4-((4-Pentylbenzoyl)oxy)-2-methoxybenzoic acid

Off-white solid. Yield: 1.30 g, 59.0 %. RF: 0.135 (100 % dichloromethane).

T_{CrI} 124 °C T_{NI} (73 °C)

ν_{max}/cm^{-1}: 2947, 2863, 1740, 1663, 1605, 1577, 1500, 1466, 1454, 1430, 1403, 1294, 1240, 1187, 1178, 1158, 1135, 1106, 1056, 1029 1015, 941, 870, 834, 789, 774, 752, 728, 696, 663, 640, 631, 619, 598, 558, 518, 489, 462, 427, 405

δ_H/ppm (400 MHz, DMSO-d_6): 12.68 (1 H, s, OH), 8.05 (2 H, d, J 8.4 Hz, Ar-H), 7.75 (1 H, d, J 8.4 Hz, Ar-H), 7.43 (2 H, d, J 8.4 Hz, Ar-H), 7.11 (1 H, d, J 2.1 Hz, Ar-H), 6.92 (1 H, dd, J 8.4 Hz, 2.1 Hz, Ar-H), 3.81 (3 H, s, O-$\underline{CH_3}$), 2.69 (2 H, t, J 7.6 Hz, Ar-$\underline{CH_2}$-CH_2-), 1.61 (2 H, tt, J 7.6 Hz, 7.3 Hz, Ar-CH_2-$\underline{CH_2}$-CH_2-), 1.29 (4 H, m, Ar-CH_2-CH_2-$\underline{CH_2}$-$\underline{CH_2}$-CH_3), 0.86 (3 H, t, J 7.0 Hz, Ar-CH_2-CH_2-CH_2-CH_2-$\underline{CH_3}$)

δ_C/ppm (100 MHz, DMSO-d_6): 166.65, 164.22, 159.49, 154.38, 149.46, 131.93, 130.03, 128.98, 126.20, 118.71, 113.65, 106.86, 56.16, 35.15, 30.84, 30.31, 21.97, 13.97

5.6 4-((4-Hexylbenzoyl)oxy)-2-methoxybenzoic acid

Brown solid. Yield: 1.46 g, 58.1 %. RF: 0.206 (100 % dichloromethane).

T_{CrI} 106 °C T_{NI} (63 °C)

ν_{max}/cm^{-1}: 2949, 2925, 2852, 1730, 1679, 1603, 1582, 1494, 1466, 1414, 1323, 1299, 1236, 1190, 1174, 1158, 1141, 1096, 1058, 1031, 1017, 949, 891, 875, 848, 828, 791, 773, 759, 739, 726, 696, 663, 628, 596, 554, 526, 463, 443, 407

δ_H/ppm (400 MHz, DMSO-d_6): 12.72 (1 H, s, OH), 8.05 (2 H, d, J 8.3 Hz, Ar-H), 7.75 (1 H, d, J 8.4 Hz, Ar-H), 7.44 (2 H, d, J 8.3 Hz, Ar-H), 7.12 (1 H, d, J 2.1 Hz, Ar-H), 6.93 (1 H, dd, J 8.4 Hz, 2.1 Hz, Ar-H), 3.82 (3 H, s, O-$\underline{CH_3}$), 2.70 (2 H, t, J 7.6 Hz, Ar-$\underline{CH_2}$-CH_2-), 1.61 (2 H, tt, J 7.6 Hz, 7.0 Hz, Ar-CH_2-$\underline{CH_2}$-CH_2-), 1.28 (6 H, m, Ar-CH_2-CH_2-$\underline{CH_2}$-$\underline{CH_2}$-$\underline{CH_2}$-CH_3), 0.86 (3 H, t, J 7.0 Hz, Ar-CH_2-CH_2-CH_2-CH_2-CH_2-$\underline{CH_3}$)

δ_C/ppm (100 MHz, DMSO-d_6): 166.66, 164.23, 159.49, 154.39, 149.47, 131.94, 130.04, 128.98, 126.20, 118.70, 113.66, 106.86, 56.16, 35.20, 31.11, 30.60, 28.29, 22.11, 14.01

(3-Fluoro-4-nitrophenyl) 2-methoxy-4-((4-alkylbenzoyl)oxy)benzoates (6)

To a pre-dried flask flushed with argon, **Compound 5** (1 eq), 3-fluoro-4-nitrophenol (1.2 eq), and *N,N'*-dicyclohexylcarbodiimide (1.5 eq) were added to the flask. The solids were solubilised with dichloromethane (30 mL) and stirred for 30 min before 4-dimethylaminopyridine (0.15 eq) was added. The quantities of the reagents used in each reaction are listed in **Table S6**. The temperature of the reaction mixture was increased to room temperature and the reaction was allowed to proceed overnight. For the reactions with *N,N'*-dicyclohexylcarbodiimide, the white precipitate which formed was removed by vacuum filtration and the filtrate collected. The solvent was removed under vacuum and the crude product was purified using a silica gel column with an appropriate solvent system (RF values quoted in product data). The eluent fractions of interest were evaporated under vacuum to leave a white solid which was recrystallised from hot ethanol (80 mL).

Table S6. Quantities of reagents used in the syntheses of the (3-fluoro-4-nitrophenyl) 2-methoxy-4-((4-alkylbenzoyl)oxy)benzoates (**6**)

n	(**5**)	3-Fluoro-4-nitrophenol	4-Dimethylaminopyridine	*N,N′*-Dicyclohexylcarbodiimide
1	0.300 g, 1.06×10^{-3} mol	0.198 g, 1.26×10^{-3} mol	19 mg, 1.58×10^{-4} mol	0.326 g, 1.58×10^{-3} mol
2	0.300 g, 9.99×10^{-4} mol	0.189 g, 1.20×10^{-3} mol	18 mg, 1.50×10^{-4} mol	0.309 g, 1.50×10^{-3} mol
3	0.300 g, 9.54×10^{-4} mol	0.179 g, 1.14×10^{-3} mol	17 mg, 1.43×10^{-4} mol	0.295 g, 1.43×10^{-3} mol
4	0.300 g, 9.14×10^{-4} mol	0.173 g, 1.10×10^{-3} mol	16 mg, 1.37×10^{-4} mol	0.283 g, 1.37×10^{-3} mol
5	0.300 g, 8.76×10^{-4} mol	0.165 g, 1.05×10^{-3} mol	16 mg, 1.31×10^{-4} mol	0.270 g, 1.31×10^{-3} mol
6	0.300 g, 8.42×10^{-4} mol	0.159 g, 1.00×10^{-3} mol	15 mg, 1.26×10^{-4} mol	0.260 g, 1.26×10^{-3} mol

6.1 (3-Fluoro-4-nitrophenyl) 2-methoxy-4-((4-methylbenzoyl)oxy)benzoate (1EC6F)

Yield: 0.111 g, 24.6 %. RF: 0.216 (100 % dichloromethane).

T_{CrI} 169 °C T_{N_FI} (156 °C)

ν_{max}/cm^{-1}: 1754, 1738, 1731, 1610, 1581, 1529, 1484, 1457, 1412, 1347, 1316, 1263, 1219, 1194, 1180, 1168, 1134, 1092, 1069, 1033, 1007, 966, 908, 889, 878, 856, 837, 811, 793, 758, 744, 734, 684, 637, 614, 556, 529, 476, 455

δ_H/ppm (400 MHz, $CDCl_3$): 8.14 (4 H, m, Ar-H), 7.34 (2 H, d, J 8.2 Hz, Ar-H), 7.29 (1 H, dd, J 11.4 Hz, 2.4 Hz, Ar-H), 7.21 (1 H, m, Ar-H), 6.97 (2 H, m, Ar-H), 3.97 (3 H, s, O-$\underline{CH_3}$), 2.48 (3 H, s, Ar-$\underline{CH_3}$)

δ_F/ppm (376 MHz, $CDCl_3$): -113.37

δ_C/ppm (100 MHz, $CDCl_3$): 164.61, 162.00, 161.95, 157.68, 156.88, 156.11, 156.01, 155.03, 145.26, 134.84, 134.77, 133.94, 130.48, 129.62, 127.26, 127.25, 126.19, 118.35, 118.31, 114.70, 114.04, 112.70, 112.46, 106.60, 56.51, 21.98

MS = $[M+Na]^+$: Calculated for $C_{22}H_{16}FNO_7Na$: 448.0808. Found: 448.0814. Difference: 1.3 ppm

6.2 (3-Fluoro-4-nitrophenyl) 2-methoxy-4-((4-ethylbenzoyl)oxy)benzoate (2EC6F)

Yield: 0.173 g, 39.4 %. RF: 0.289 (100 % dichloromethane).

T_{CrI} 146 °C T_{N_FI} (132 °C)

ν_{max}/cm^{-1}: 3061, 2969, 1727, 1705, 1601, 1584, 1526, 1487, 1474, 1463, 1411, 1343, 1275, 1251, 1230, 1194, 1175, 1159, 1143, 1112, 1094, 1076, 1047, 1017, 968, 949, 907, 891, 842, 824, 755, 736, 700, 686, 665, 631, 604, 572, 547, 520, 465, 450, 423, 409

δ_H/ppm (400 MHz, $CDCl_3$): 8.14 (4 H, m, Ar-H), 7.37 (2 H, d, J 8.2 Hz, Ar-H), 7.29 (1 H, dd, J 11.4 Hz, 2.4 Hz, Ar-H), 7.22 (1 H, m, Ar-H), 6.96 (2 H, m, Ar-H), 3.97 (3 H, s, O-$\underline{CH_3}$), 2.77 (2 H, quart, 7.6 Hz, Ar-$\underline{CH_2}$-CH_3), 1.30 (3 H, t, 7.6 Hz, Ar-CH_2-$\underline{CH_3}$)

δ_F/ppm (376 MHz, $CDCl_3$): -113.38

δ_C/ppm (100 MHz, $CDCl_3$): 164.63, 161.98, 161.94, 157.66, 156.88, 156.09, 155.99, 155.01, 149.92, 134.80, 134.73, 133.95, 130.49, 129.04, 127.27, 127.25, 126.36, 118.36, 118.32, 114.63, 114.03, 112.70, 112.47, 106.55, 56.49, 29.24, 15.35

MS = $[M+Na]^+$: Calculated for $C_{23}H_{18}FNO_7$: 462.0965. Found: 462.0947. Difference: 3.9 ppm

6.3 (3-Fluoro-4-nitrophenyl) 2-methoxy-4-((4-propylbenzoyl)oxy)benzoate (3EC6F)

Yield: 0.196 g, 45.3 %. RF: 0.472 (100 % dichloromethane).

T_{CrI} 150 °C $T_{N_FN_X}$ (116 °C) T_{N_XN} (118 °C) T_{NI} (123 °C)

ν_{max}/cm^{-1}: 3061, 2962, 2928, 1727, 1705, 1602, 1583, 1527, 1488, 1473, 1410, 1344, 1275, 1251, 1231, 1194, 1178, 1158, 1113, 1094, 1078, 1052, 1018, 969, 948, 908, 891, 869, 841, 824, 753, 740, 700, 686, 670, 635, 609, 573, 546, 515, 460, 428, 407

δ_H/ppm (400 MHz, $CDCl_3$): 8.18 (1 H, t, J 8.8 Hz, Ar-H), 8.12 (3 H, m, Ar-H), 7.35 (2 H, d, J 8.4 Hz, Ar-H), 7.28 (1 H, dd, J 11.4 Hz, 2.4 Hz, Ar-H), 7.21 (1 H, m, Ar-H), 6.96 (2 H, m, Ar-H), 3.97 (3 H, s, O-$\underline{CH_3}$), 2.70 (2 H, t, J 7.4 Hz, Ar-$\underline{CH_2}$-CH_2-), 1.70 (2 H, m, Ar-CH_2-$\underline{CH_2}$-CH_3), 0.97 (3 H, t, J 7.2 Hz, Ar-CH_2-CH_2-$\underline{CH_3}$)

δ_F/ppm (376 MHz, $CDCl_3$): -113.34

δ_C/ppm (100 MHz, $CDCl_3$): 164.63, 161.98, 161.95, 157.66, 156.88, 156.09, 155.99, 155.01, 149.92, 134.80, 134.73, 133.95, 130.49, 129.04, 127.27, 127.25, 126.36, 118.36, 118.32, 114.63, 114.03, 112.70, 112.47, 106.58, 56.49, 38.27, 24.39, 13.89

MS = $[M+H]^+$: Calculated for $C_{24}H_{21}FNO_7$: 454.1302. Found: 454.1311. Difference: 2.0 ppm

6.4 (3-Fluoro-4-nitrophenyl) 2-methoxy-4-((4-butylbenzoyl)oxy)benzoate (4EC6F)

Yield: 0.210 g, 56.3 %. RF: 0.400 (100 % dichloromethane).

T_{CrI} 120 °C $T_{N_FN_X}$ (79 °C) T_{N_XN} (92 °C) T_{NI} (105 °C)

ν_{max}/cm^{-1}: 3062, 2959, 2931, 2857, 1727, 1706, 1601, 1585, 1526, 1487, 1474, 1462, 1413, 1343, 1275, 1253, 1230, 1195, 1178, 1159, 1143, 1114, 1093, 1075, 1054, 1017, 967, 950, 906, 891, 841, 826, 753, 700, 685, 671, 654, 635, 610, 592, 573, 547, 517, 464, 423, 408

δ_H/ppm (400 MHz, $CDCl_3$): 8.18 (1 H, t, J 8.9 Hz, Ar-H), 8.12 (3 H, m, Ar-H), 7.35 (2 H, d, J 8.4 Hz, Ar-H), 7.29 (1 H, dd, J 11.4 Hz, 2.4 Hz, Ar-H), 7.22 (1 H, m, Ar-H), 6.96 (2 H, m, Ar-H), 3.97 (3 H, s, O-$\underline{CH_3}$), 2.72 (2 H, t, J 7.7 Hz, Ar-$\underline{CH_2}$-CH_2-), 1.65 (2 H, m, Ar-CH_2-$\underline{CH_2}$-CH_2-), 1.38 (2 H, m, Ar-CH_2-CH_2-$\underline{CH_2}$-CH_3), 0.95 (3 H, t, J 7.3 Hz, Ar-CH_2-CH_2-CH_2-$\underline{CH_3}$)

δ_F/ppm (376 MHz, $CDCl_3$): -113.34

δ_C/ppm (100 MHz, $CDCl_3$): 164.63, 161.98, 161.95, 157.66, 156.88, 156.09, 155.99, 155.01, 150.18, 134.80, 134.73, 133.95, 130.50, 128.98, 127.27, 127.25, 126.31, 118.36, 118.32, 114.63, 114.04, 112.70, 112.47, 106.58, 56.49, 35.96, 33.38, 22.46, 14.06

MS = $[M+H]^+$: Calculated for $C_{25}H_{23}FNO_7$: 468.1459. Found: 468.1468. Difference: 1.9 ppm

6.5 (3-Fluoro-4-nitrophenyl) 2-methoxy-4-((4-pentylbenzoyl)oxy)benzoate (5EC6F)

Yield: 0.193 g, 45.8 %. RF: 0.474 (100 % dichloromethane).

T_{CrI} 114 °C T_{N_XN} (85 °C) T_{NI} (109 °C)

ν_{max}/cm^{-1}: 3062, 2958, 2927, 2854, 1729, 1706, 1602, 1584, 1527, 1488, 1474, 1412, 1343, 1275, 1253, 1231, 1194, 1177, 1159, 1114, 1094, 1073, 1054, 1023, 968, 949, 906, 891, 864, 841, 825, 750, 728, 700, 686, 671, 646, 635, 610, 573, 547, 516, 481, 465, 429, 420, 409

δ_H/ppm (400 MHz, $CDCl_3$): 8.18 (1 H, t, J 9.0 Hz, Ar-H), 8.12 (3 H, m, Ar-H), 7.34 (2 H, d, J 8.4 Hz, Ar-H), 7.29 (1 H, dd, J 11.4 Hz, 2.4 Hz, Ar-H), 7.21 (1 H, m, Ar-H), 6.96 (2 H, m, Ar-H), 3.97 (3 H, s, O-$\underline{CH_3}$), 2.71 (2 H, t, J 7.5 Hz, Ar-$\underline{CH_2}$-CH_2-), 1.67 (2 H, m, Ar-CH_2-$\underline{CH_2}$-CH_2-), 1.35 (4 H, m, Ar-CH_2-CH_2-$\underline{CH_2}$-$\underline{CH_2}$-CH_3), 0.91 (3 H, t, J 6.9 Hz, Ar-CH_2-CH_2-CH_2-CH_2-$\underline{CH_3}$)

δ_F/ppm (376 MHz, $CDCl_3$): -113.33

δ_C/ppm (100 MHz, $CDCl_3$): 164.63, 161.98, 161.95, 157.67, 156.89, 156.10, 155.99, 155.02, 150.21, 134.81, 134.74, 133.95, 130.51, 128.99, 127.27, 127.25, 126.31, 118.36, 118.33, 114.63, 114.04, 112.71, 112.47, 106.58, 56.49, 36.24, 31.56, 30.95, 22.65, 14.16

MS = $[M+H]^+$: Calculated for $C_{26}H_{25}FNO_7$: 482.1615. Found: 482.1636. Difference: 4.4 ppm

6.6 (3-Fluoro-4-nitrophenyl) 2-methoxy-4-((4-hexylbenzoyl)oxy)benzoate (6EC6F)

Yield: 0.169 g, 40.5 %. RF: 0.314 (100 % dichloromethane).

T_{CrI} 104 °C T_{N_XN} (69 °C) T_{NI} (97 °C)

ν_{max}/cm^{-1}: 3036, 2927, 2853, 1728, 1708, 1602, 1585, 1527, 1487, 1474, 1412, 1344, 1309, 1276, 1254, 1229, 1195, 1176, 1157, 1143, 1113, 1094, 1073, 1054, 1024, 1016, 967, 949, 906, 890, 851, 843, 823, 752, 702, 686, 671, 644, 636, 611, 594, 572, 547, 517, 464, 439, 421, 408

δ_H/ppm (400 MHz, $CDCl_3$): 8.18 (1 H, t, J 8.7 Hz, Ar-H), 8.12 (3 H, m, Ar-H), 7.34 (2 H, d, J 8.3 Hz, Ar-H), 7.29 (1 H, dd, J 11.4 Hz, 2.4 Hz, Ar-H), 7.21 (1 H, m, Ar-H), 6.96 (2 H, m, Ar-H), 3.79 (3 H, s, O-$\underline{CH_3}$), 2.72 (2 H, t, J 7.7 Hz, Ar-$\underline{CH_2}$-CH_2-), 1.65 (2 H, m, Ar-CH_2-$\underline{CH_2}$-CH_2-), 1.33 (6 H, m, Ar-CH_2-CH_2-$\underline{CH_2}$-$\underline{CH_2}$-$\underline{CH_2}$-CH_3), 0.90 (3 H, t, J 6.8 Hz, Ar-CH_2-CH_2-CH_2-CH_2-CH_2-$\underline{CH_3}$)

δ_F/ppm (376 MHz, $CDCl_3$): -113.37

δ_C/ppm (100 MHz, $CDCl_3$): 164.62, 161.99, 161.95, 157.67, 156.90, 156.11, 156.01, 155.02, 150.21, 134.83, 134.76, 133.94, 130.51, 128.98, 127.26, 127.24, 126.34, 118.35, 118.31, 114.68, 114.04, 112.69, 112.46, 106.60, 56.49, 36.27, 31.79, 31.21, 29.05, 22.72, 14.22

MS = $[M+H]^+$: Calculated for $C_{27}H_{27}FNO_7$: 496.1772. Found: 496.1771. Difference: 0.2 ppm

Additional Experimental Data

Table S7. The phase transition temperatures for the *n*EC6F series with an alkyl (C*n*) terminal chain, the temperatures are given in °C and associated transition enthalpy changes (in parenthesis) in Jg^{-1}

n	m.p.	Phase Sequence
1	169.0(97.0)	N_F-155.8(11.6)-Iso
2	145.9(90.6)	N_F-132.1(9.3)-Iso
3	150.0(106.2)	N_F-116-N_X-118.0(0.8)-N-122.8(2.4)-Iso
4	120.2(78.6)	N_F-79.0(0.6)-N_X-92.2(0.03)-N-105.4(2.0)-Iso
5	113.8(76.4)	N_X-84.9(0.02)-N-109.4(2.2)-Iso
6	104.2(59.4)	N_X-68.5(0.02)-N-97.2(1.4)-Iso

Table S8. The phase transition temperatures for the *n*OEC3F series with an alkyloxy (OC*n*) terminal chain, the temperatures are given in °C and associated transition enthalpy changes (in parenthesis) in Jg^{-1}

n	m.p.	Phase Sequence
1	179.0(113.4)	N_F-142.4(2.4)-N-156.8(2.0)-Iso
2	164.0(103.4)	N_F-104.2(0.6)-N_X-117.9(0.03)-N-152.5(2.4)-Iso
3	144.9(90.2)	N_X-79.0(0.01)-N-133.3(1.4)-Iso
4	127.8(87.7)	N_X-38-N-130.3(1.5)-Iso
5	91.9(62.5)	N-118.0(1.1)-Iso

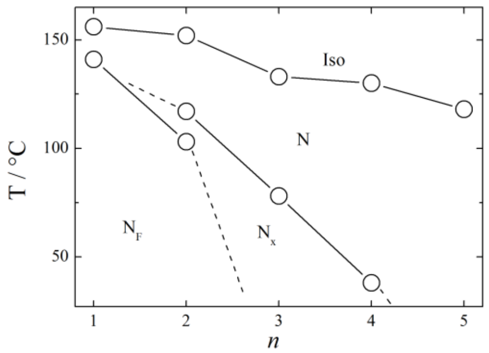

Figure S1. Phase diagram for studied homologue series of ferronematogens with alkyloxy terminal chain, *n*, molecular formula given above.

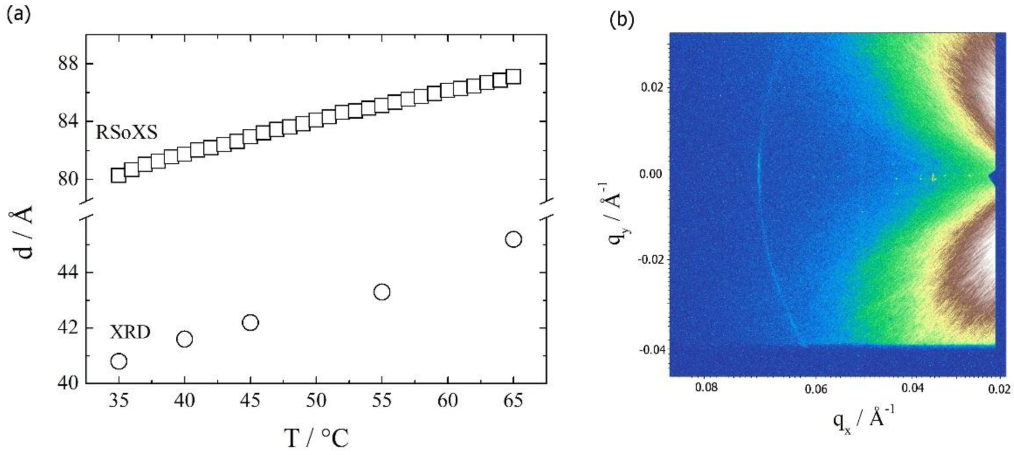

Figure S2. (a) Comparison of the periodicities of the antiferroelectric domain structure in the N_X phase for the *3*OEC6F measured using non-resonant (XRD) and resonant (RSoXS) X-ray diffraction methods; (b) the 2D RSoXS pattern registered at T = 65 °C, increased background at low q-values is due to strong anisotropic scattering on a TEM grid used as a sample holder.

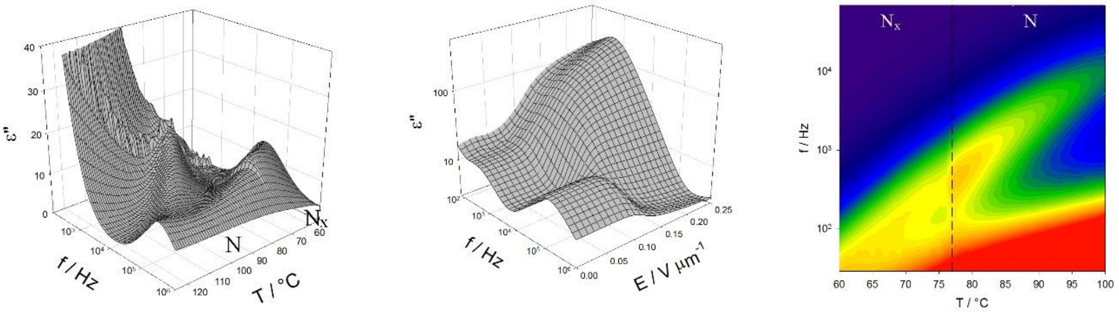

Figure S3. The Imaginary part of the dielectric susceptibility measured for homologue 3OEC3F: (left) the temperature and frequency dependence across the N-N_X phase sequence, (middle) the frequency and bias field dependence in the N_X phase (at T = 73 °C); where the high-frequency mode is suppressed and the lower-frequency 'ferroelectric' mode is excited above threshold field of 0.11 V/µm; and (right) a map showing evolution the 'ferroelectric' mode vs. temperature and frequency, the measurements were performed under bias electric field 0.3 V/µm.

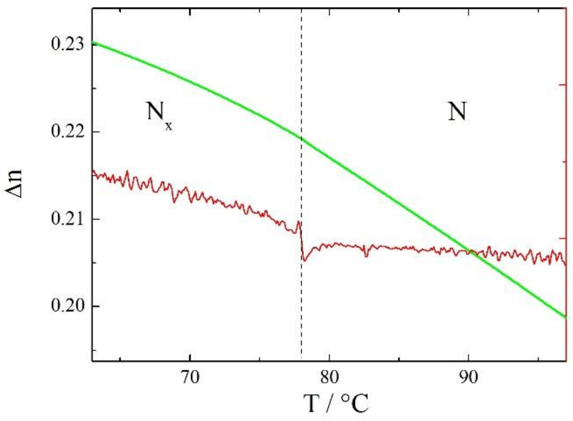

Figure S4. Optical birefringence (green line) of 3OEC3F measured with green light (λ = 532 nm) across the N-N_X phase transition. The red line shows derivative d(Δn)\dT, evidencing the phase transition temperature.

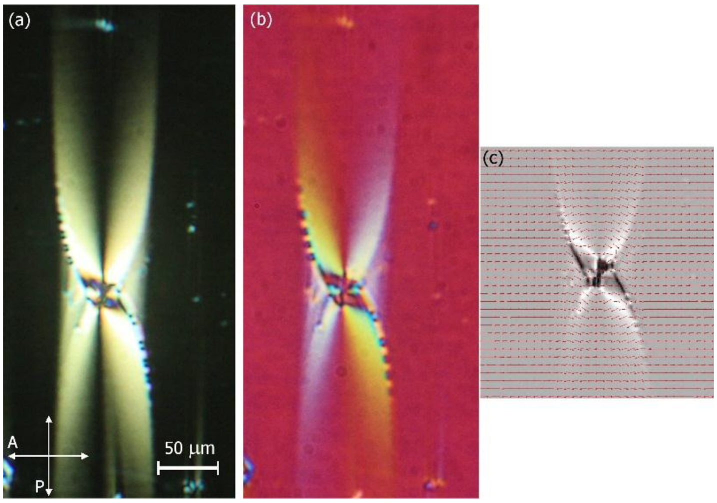

Figure S5. Optical texture with focal conic-twin like defects in N_F phase taken for 4EC6F in 1.6-μm-thick cell with planar anchoring: (a) between crossed polarizers, (b) with λ retardation plate inserted at 45 deg. with respect to polarizers, (c) the optical retardation and director field determined with Abrio system (at λ = 546 nm). The defects are anchored on rods used as separators in glass cells.
